# *Sesamum indicum* Oleosin L improves oil packaging in *Nicotiana benthamiana* leaves

**DOI:** 10.1002/pld3.343

**Published:** 2021-09-06

**Authors:** Suyan Yee, Vivien Rolland, Kyle B. Reynolds, Pushkar Shrestha, Lina Ma, Surinder P. Singh, Thomas Vanhercke, James R. Petrie, Anna El Tahchy

**Affiliations:** ^1^ Commonwealth Scientific and Industrial Research Organisation, Agriculture and Food Acton ACT Australia; ^2^ Research School of Biology The Australian National University Canberra ACT Australia

**Keywords:** leaf oil, lipid biosynthesis, lipid droplet protein, oil increase, oleosin, *Nicotiana benthamiana*, triacylglycerol storage

## Abstract

Plant oil production has been increasing continuously in the past decade. There has been significant investment in the production of high biomass plants with elevated oil content. We recently showed that the expression of *Arabidopsis thaliana* WRI1 and DGAT1 genes increase oil content by up to 15% in leaf dry weight tissue. However, triacylglycerols in leaf tissue are subject to degradation during senescence. In order to better package the oil, we expressed a series of lipid droplet proteins isolated from bacterial and plant sources in *Nicotiana benthamiana* leaf tissue. We observed further increases in leaf oil content of up to 2.3‐fold when we co‐expressed *Sesamum indicum* Oleosin L with *At*WRI1 and *At*DGAT1. Biochemical assays and lipid droplet visualization with confocal microscopy confirmed the increase in oil content and revealed a significant change in the size and abundance of lipid droplets.

## 1 INTRODUCTION

Plant triacylglycerols (TAGs), more commonly known as vegetable oils, have a variety of uses ranging from human consumption to biodiesel production (Durrett et al., 2008). Studies have shown that the demand for plant oils for both food and non‐food purposes have increased with the rising global population (Gunstone, 2011) and will continue to increase in the next decade (Chapman & Ohlrogge, 2012).

In oilseed plants like *Sesamum indicum*, storage oil and proteins accumulate during seed maturation within dynamic subcellular compartments called protein storage vacuoles (PSVs) and oil bodies (OBs), the latter also being referred to as oleosomes and lipid droplets (LDs) in plant seeds and non‐plant organisms (Deruyffelaere et al., 2015). OBs and LDs are approximately .5 to 2 μm in diameter and consist of a sphere‐like core matrix of neutral lipids, such as TAGs and sterol esters, which are surrounded by a monolayer of amphipathic lipids, primarily phospholipids and sterols (Huang, 2018). The small size of OBs and LDs provides a large surface area‐to‐volume ratio of lipid monolayer per unit of TAG, and generally contain specific membrane‐associated proteins such as oleosins to facilitate lipase binding and lipolysis during germination (Chapman & Ohlrogge, 2012; Deruyffelaere et al., 2015; Hsieh & Huang, 2004; Huang & Huang, 2016; Murphy, 2012).

Oleosins are a family of oil body‐membrane proteins that function in proper OB and LD formation and stabilization for long‐term lipid storage (Shimada & Hara‐Nishimura, 2010). Oleosins are generally made up of an N‐terminal domain, a central hydrophobic domain, and a C‐terminal domain (Hsiao & Tzen, 2011). Oleosins are typically not expressed in leaf tissue (Vanhercke et al., 2014), and are instead predominantly expressed in seeds (Jolivet et al., 2004). Lipid reserves are metabolized via the successive events of lipolysis, fatty acid (FA) transport to glyoxysomes, activation of acyl‐CoA derivatives, β‐oxidation, the glyoxylate cycle, the partial tricarboxylic acid cycle, and gluconeogenesis (Deruyffelaere et al., 2015).

Recent proteomics‐ and homology‐based studies have also led to the identification of several new protein components involved in the formation, maintenance, function and turnover of LDs, specifically in non‐seed plant tissue (Pyc et al., 2017; Reynolds et al., 2019; Zhi et al., 2017). These protein components include small rubber particle proteins (SRPPs) isolated from *Persea americana* (avocado) mesocarp LDs, caleosins, and LD‐associated proteins (LDAPs) in *Arabidopsis thaliana* (Horn et al., 2013). There have also been a number of other proteins found to be frequently associated with LDs in the seeds of plants, including proteases, phospholipases, lipoxygenases, and lipases (Chapman et al., 2012; Rudolph et al., 2011).

Given the breakthroughs in elucidating and manipulating the lipid biosynthesis pathway in plants over the last 10 years, producing oil in the vegetative tissues of high biomass crops has never been a more attractive alternative to the conventional approach of producing oil in seeds. Several parts of the plant lipid biosynthesis pathway have been successfully targeted using various approaches. Strategies with a focus on down‐regulating or overexpressing single genes or multiple gene combinations involved in FA and TAG metabolism have been investigated (Dong et al., 2014; Eastmond, 2006; Meyer et al., 2012; Reynolds et al., 2015; Vanhercke et al., 2013; Vanhercke et al., 2014; Vanhercke et al., 2019; Zhang et al., 2018). Examples of these approaches include the upregulation of lipid biosynthesis by overexpressing transcription factors found in seed tissue, like LEAFY COTYLEDON2 (LEC2) and WRINKLED1 (WRI1), upregulating oil accumulation pathways via diacylglycerol acyltransferase (DGAT) overexpression, and minimizing lipase‐mediated catabolism of seed oil through the silencing of TAG lipases, such as SUGAR‐DEPENDENT LIPASE1 (SDP1) (Ohlrogge & Chapman, 2011; Eastmond, 2006; Kong & Ma, 2018; Baud et al., 2009; Kim et al., 2012; Vanhercke et al., 2019). Such metabolic engineering strategies rely on the simultaneous “push” to favor the production of FAs through the upregulation of WRI1, the “pull” towards conversion into TAGs via DGAT1, and the “protection” of newly synthesized TAGs from degradation or oxidation by downregulating breakdown pathways and/or expressing protective LD coat proteins, like OLEOSIN during storage (Vanhercke et al., 2014).

Several studies have focused their attention on WRI1 and DGAT1, as their roles in the biosynthetic pathway of plant oils have been well characterized. Similarly, these critical genes have been shown to impart a significant synergistic TAG accumulating effect when co‐expressed in various plant models and crops (El Tahchy et al., 2017; Liu et al., 2017; Vanhercke et al., 2017; Vanhercke et al., 2019). The WRI1 transcription factor is a key transcriptional regulator of FA biosynthesis in both seed and non‐seed tissue (Baud et al., 2009; Cernac & Benning, 2004; Deng et al., 2019; Kong & Ma, 2018; Ma et al., 2013), whereas DGAT1 acts as a gate keeper to the committed metabolic step towards TAG production by catalyzing the conversion of diacylglycerols and fatty acyl CoA substrates into TAGs (Guo et al., 2017; Ståhl et al., 2004).

Various groups have also demonstrated the capacity to stably accumulate these TAGs in non‐seed tissue of a variety of species, such as *Nicotiana tabacum*, *Nicotiana benthamiana*, *Sorghum bicolor*, and *Solanum tuberosum* (potato) tubers through the co‐expression of *At*WRI1, *At*DGAT1 and an intron‐interrupted oleosin from sesame (El Tahchy et al., 2017; Liu et al., 2017; Vanhercke et al., 2017; Vanhercke et al., 2019). However, few studies have investigated the effects of other *S. indicum* oleosin isoforms on the accumulation and stability of LDs in leaf tissue. Similarly, lipid droplet proteins from other high oil‐producing plants, such as *P. americana* (avocado) and *Vanilla planifolia* (vanilla) have rarely been examined as potential protein targets to enhance TAG accumulation, as well as to improve LD stability in transient transgenic systems.

In this study, we hypothesized that screening a number of LD proteins from plant and bacterial sources could unravel new protein targets with significant TAG accumulating properties, as well as new engineering opportunities to further improve TAG accumulation and lipid packaging in plant leaf tissue. We selected five proteins of interest, based on the important roles they play in lipid packaging in their respective source organisms: two *S. indicum* oleosin isoforms (L and H), the *V. planifolia* leaf oleosin U1, the *P. americana* mesocarp oleosin M and the *Rhodococcus opacus* TadA LD protein. We used a transient expression system in *N. benthamiana* to test the effect of these five genes on TAG accumulation when combined with the positive synergistic effects of *At*WRI1 and *At*DGAT1 (Vanhercke et al., 2013). We report that *Si*Oleosin L led to the accumulation of small LDs and a significant increase in TAG yields resulting in efficient and stable TAG packaging in *N. benthamiana* leaves.

## 2 MATERIALS AND METHODS

### 2.1 Assembly of expression vectors

The gene encoding the bacterial *R. opacus* TadA LD protein (MacEachran et al., 2010) (GenBank: HM625859) was codon optimized for expression in *N. benthamiana* before being synthesized as a *Not*I‐*Spe*I fragment via the GeneArt gene synthesis platform (Thermo Fisher Scientific – AU). The *TadA* gene fragment was subsequently cloned downstream of the 35S promoter in the binary vector, pJP3343 (Reynolds et al., 2015) using the *Not*I‐*Spe*I restriction sites. This resulting plasmid was designated pOIL380.

The genes coding for the *S. indicum* Oleosin isoform L (Tai et al., 2002) (GenBank: AF091840) and the *S. indicum* Oleosin isoform H1 LD proteins (Tai et al., 2002) (GenBank: AF302807) were also codon optimized for *N. benthamiana* expression and synthesized as *Not*I‐*Sac*I fragments through the GeneArt gene synthesis platform (ThermoFisher Scientific – AU). These synthesized gene fragments were subsequently cloned downstream of the 35S promoter in pJP3343 using the *Not*I*‐Sac*I restriction sites. The resulting plasmids were designated pOIL382 and pOIL383, respectively.

Finally, the genes encoding the *V. planifolia* leaf Oleosin U1 (Huang & Huang, 2016) (GenBank: SRX648194) and the *P. americana* mesocarp Oleosin M lipid droplet protein (Huang & Huang, 2016) (GenBank: SRX627420) were codon optimized for expression in *N. benthamiana*, synthesized as *Eco*RI‐*Spe*I fragments via the GeneArt gene synthesis service (Thermo Fisher Scientific – AU), and subsequently cloned downstream of the 35S promoter using the respective restriction sites in the binary vector, pJP3343. These resulting plasmids were then designated pOIL386 and pOIL387, respectively.

Binary expression vectors containing *At*WRI1 and *At*DGAT1 were previously described (Vanhercke et al., 2013). Each of these expression constructs were transformed into *Agrobacterium tumefaciens* strain AGL1 for transient expression in plant tissue.

Assembled plasmid maps and sequences of pOIL380, pOIL382, pOIL383, pOIL386 and pOIL387 containing their respective codon‐optimized oleosin genes are supplied as Supplementary Materials.

### 2.2 Transient expression assays in *N. benthamiana*

Transient expression in *N. benthamiana* leaves was performed as previously described (Wood et al., 2009), with some minor modifications. Specifically, *A. tumefaciens* AGL1 cultures containing the plasmids coding for the p19 viral suppressor protein and the LD gene(s) of interest were mixed such that the final OD_600_ of each culture was equal to .125 prior to infiltration (Vanhercke et al., 2013). Samples being compared were randomly located on the same leaf. Infiltrations for each sample were repeated across three different leaves located on independent plants, acting as separate biological replicates. The infiltrated *N. benthamiana* plants were then grown for a further four days before leaf discs were harvested. For biochemical analyses, leaf discs were harvested and pooled from across the three infiltrated leaves, freeze‐dried, weighed and stored at −80 °C. For confocal microscopy analyses, fresh leaf discs from the same infiltration spot as those used for biochemical analyses were imaged within 30–45 min of harvesting (see below).

### 2.3 Lipid extraction and analysis

Total lipids were extracted from freeze‐dried *N. benthamiana* tissue four days post‐infiltration (dpi) using chloroform:methanol: .1 M KCl (2:1:1 v/v/v), as described previously (El Tahchy et al., 2017). Fatty acid methyl esters (FAME) were then analyzed via gas chromatography techniques, specifically via a GC‐FID (7890A GC, Agilent Technologies, Palo Alto, CA) equipped with a 30 m BPX70 column (.25 mm inner diameter, .25 mm film thickness, SGE, Austin, USA), as described previously (El Tahchy et al., 2017; Petrie et al., 2012). Resultant peaks were integrated with the Agilent Technologies ChemStation software (Rev B.04.03).

### 2.4 Confocal imaging of lipid droplets

Lipid droplets located in the spongy mesophyll of fresh *N. benthamiana* leaf discs were imaged four days post‐infiltration, as follows. The abaxial epidermis was peeled off in 50 mM PIPES pH 7.0 immediately after collection and discarded. One half of each peeled disc was stained for 10 min in 2 μg/ml BODIPY505/515 in 50 mM PIPES pH 7.0, followed by 2–3 washes in 50 mM PIPES pH 7.0. During this time, the other half of the leaf disc was kept in 50 mM PIPES pH 7.0. Peeled leaf discs were then mounted in 50 mM PIPES pH 7.0 and imaged immediately, using a Leica SP8 Laser‐Scanning Confocal Microscope (Leica Microsystems AG, Germany), a 20x objective (NA = .75), and the Leica LAS X software (Leica Microsystems AG, Germany). Lipid droplets and chloroplasts were imaged by exciting leaf discs with a 505 nm laser. BODIPY 505/515 signal was collected between 510 and 540 nm, whereas chloroplast signal was collected between 650 nm and 690 nm. Unstained half discs were imaged with the same settings to determine tissue autofluorescence.

### 2.5 In vivo [^14^C] acetate feeding and pulse‐chase assays

A 1 μCi/μl [^14^C] acetate solution was prepared in the same infiltration buffer previously used to infiltrate the expression plasmids into *N. benthamiana*. Using this solution, .4 mM of [^14^C] acetate was infiltrated into the same leaves at four days post‐infiltration of the genes of interest, such that this reagent was in excess. At 5, 10, 15 min and 3 h post‐infiltration of [^14^C] acetate, leaf discs from across the three separately infiltrated leaves were pooled and [^14^C] acetate uptake was stopped with chloroform. Total lipid extraction and TLC (thin layer chromatography) fractionation was processed as previously described (El Tahchy et al., 2017). The TLC plate was exposed to phosphor imaging screens overnight and analyzed using a Fujifilm FLA‐5000 phosphor imager. Radiolabeled lipid spots (TAG and free fatty acids [FFA]) were measured using a Beckman‐Coulter Ready Safe liquid scintillation cocktail and Beckman‐Coulter LS 6500 Multipurpose Scintillation Counter (PerkinElmer).

### 2.6 Statistical analyses

All statistical tests, including Student's *t*‐tests, two‐way ANOVAs and two‐way MANOVAs with subsequent Dunnett's tests as post‐hoc analyses were performed using R (version 3.6.1) loaded onto R Studio (version 1.2.5001).

## 3 RESULTS

### 3.1 Effect of transiently expressed *At*WRI1, *At*DGAT1, and lipid droplet proteins on TAG and FA composition

**FIGURE 1:**
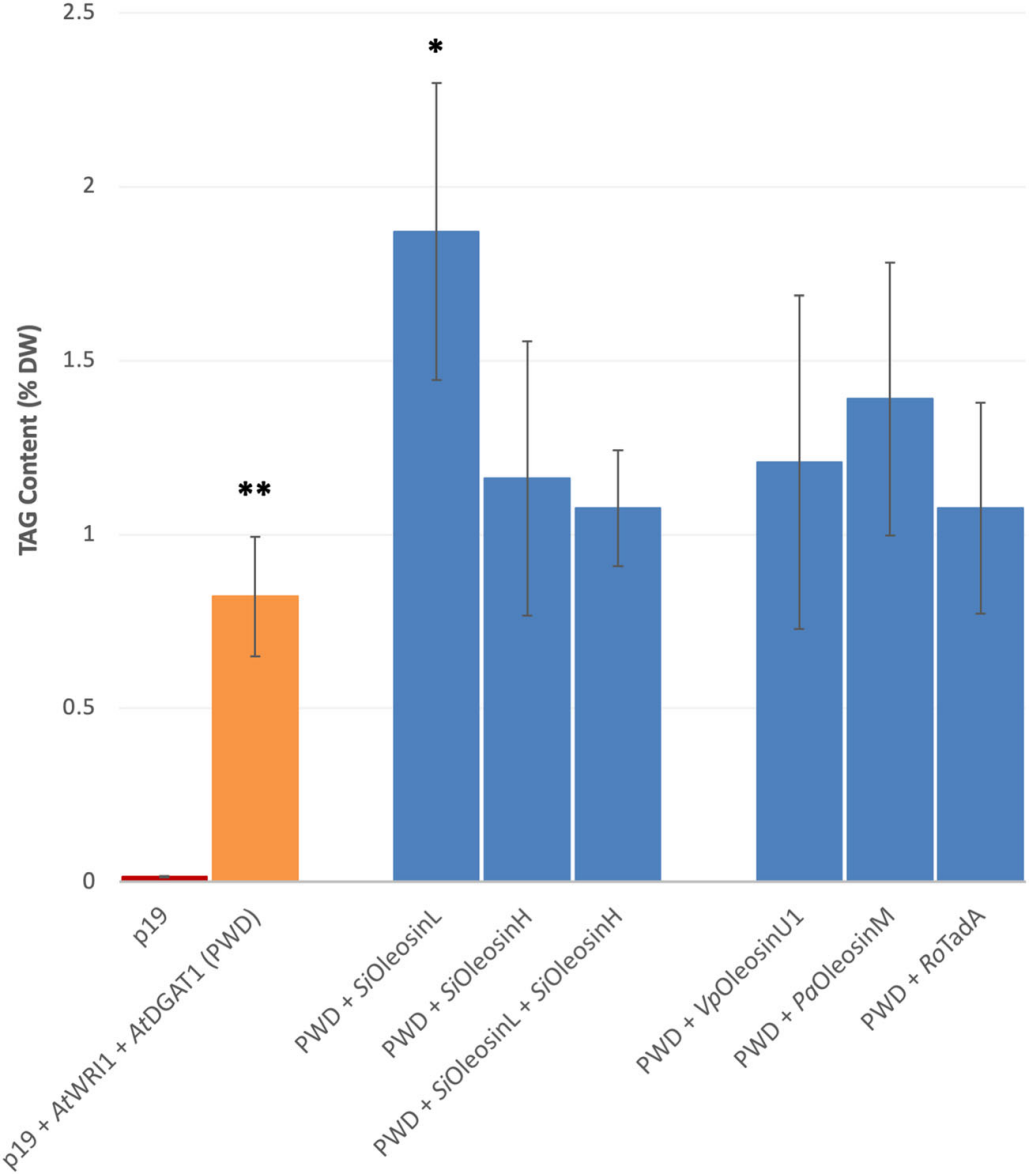
Triacylglycerol content in *Nicotiana benthamiana* leaves transiently expressing *At*WRI1, *At*DGAT1, *Si*OleosinL, *Si*OleosinH, vanilla leaf *Vp*OleosinU1, avocado mesocarp *Pa*OleosinM, and *Rhodococcus opacus*TadA lipid droplet protein. Error bars denote standard error with *n* = 3. **Significantly different at *p* < .01 against the p19 control (mean TAG content of .02% DW). *Significantly different at *p* < .01 against the p19 + *At*WRI1 + *At*DGAT1 (PWD) control

To determine which lipid droplet protein best protects lipid accumulation, we used a *N. benthamiana* transient assay to express each one of the five selected LD protein constructs in a p19 + *At*WRI1 + *At*DGAT1 (PWD) background (Figure 1). A highly significant increase in TAG content in leaves transiently expressing *Si*OleosinL was observed. This was equivalent to a 2.3‐fold increase compared with the p19 + *At*WRI1 + *At*DGAT1 (PWD) control, and a 122‐fold increase compared with the p19 only control. In contrast, no significant increase in TAG levels was observed with the co‐expression of *Si*OleosinH, *Vp*OleosinU1, *Pa*OleosinM, and *Ro*TadA lipid droplet proteins compared with the PWD background (Figure 1). Interestingly, the co‐expression of both *S. indicum* oleosin isoforms, *Si*OleosinL + *Si*OleosinH also did not result in an increase in TAG content (Figure 1).

**FIGURE 2:**
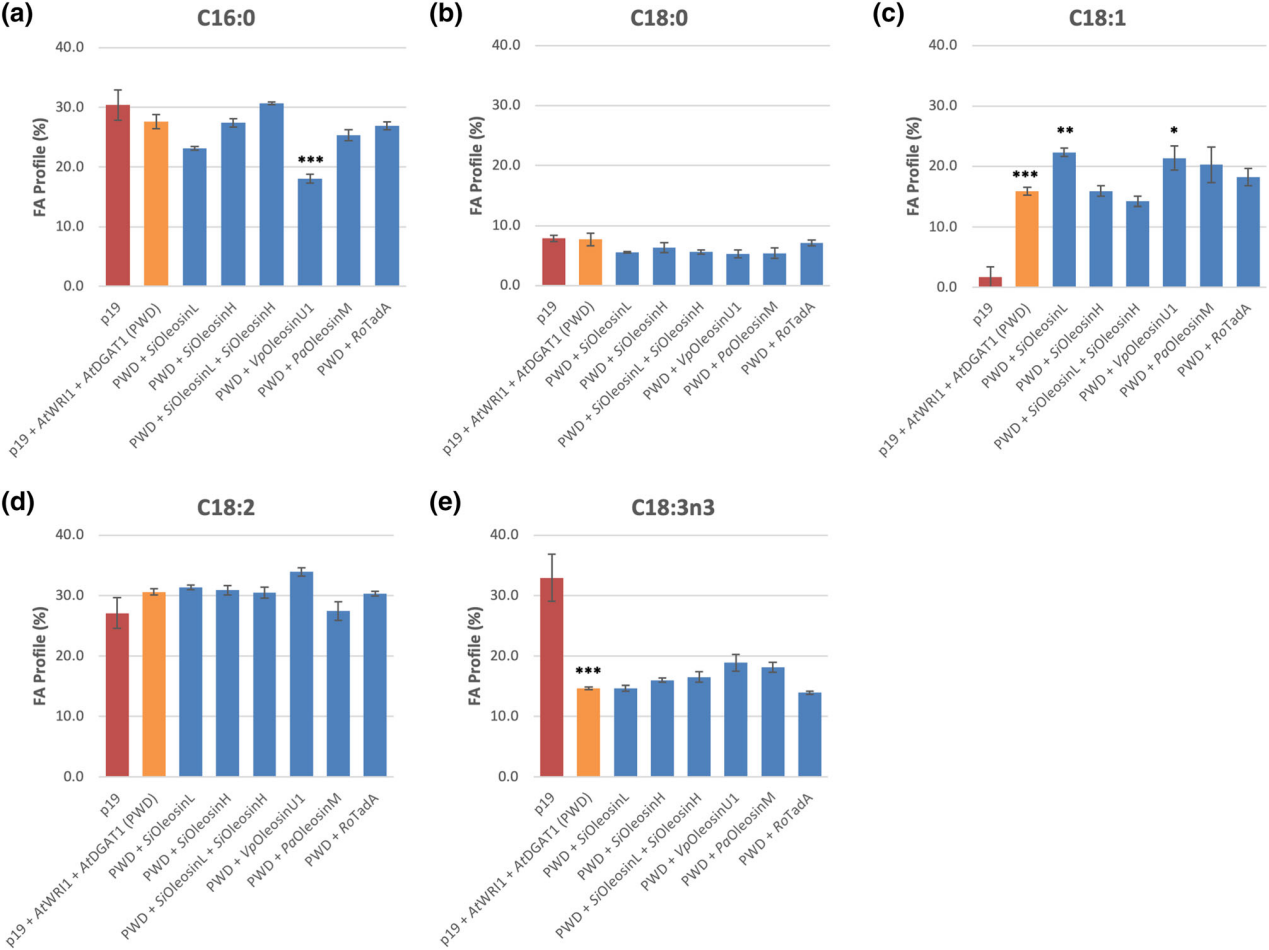
Triacylglycerol fatty acid composition, specifically (a) C16:0, (b) C18:0, (c) C18:1, (d) C18:2, and (e) C18:3n3 in *Nicotiana benthamiana* leaves transiently expressing *At*WRI1, *At*DGAT1, *Si*OleosinL, *Si*OleosinH, vanilla leaf *Vp*OleosinU1, avocado mesocarp *Pa*OleosinM, and *Rhodococcus opacus* TadA lipid droplet protein. Error bars denote standard error with *n* = 3. ***Significantly different at *p* < .001 against the p19 control. ***, **, and *Significantly different at *p* < .001, *p* < .01, and *p* < .05, respectively, against the p19 + *At*WRI1 + *At*DGAT1 (PWD) control

The significant increase in oil content observed with *Si*OleosinL was accompanied by a modification of the FA profile (Figure 2). The co‐expression of *Si*OleosinL with *At*WRI1 and *At*DGAT1 significantly increased C18:1 levels (22.3 ± .70%, Figure 2c), while decreasing C16:0 content (23.2 ± .31%, Figure 2a) compared with the PWD control, which displayed C18:1 and C16:0 levels as 15.9 ± .64% and 27.6 ± 1.16% of the total FA profile, respectively (Figure 2c and a, respectively). Interestingly, *Vp*OleosinU1 also significantly increased C18:1 levels and decreased C16:0 levels compared with the PWD control, albeit not significantly increasing overall TAG content compared with the PWD control (Figure 1). All other gene combinations tested did not show a significant effect on TAG content or FA profiles compared with the PWD control.

### 3.2 Modification of lipid droplet size and abundance

**FIGURE 3:**
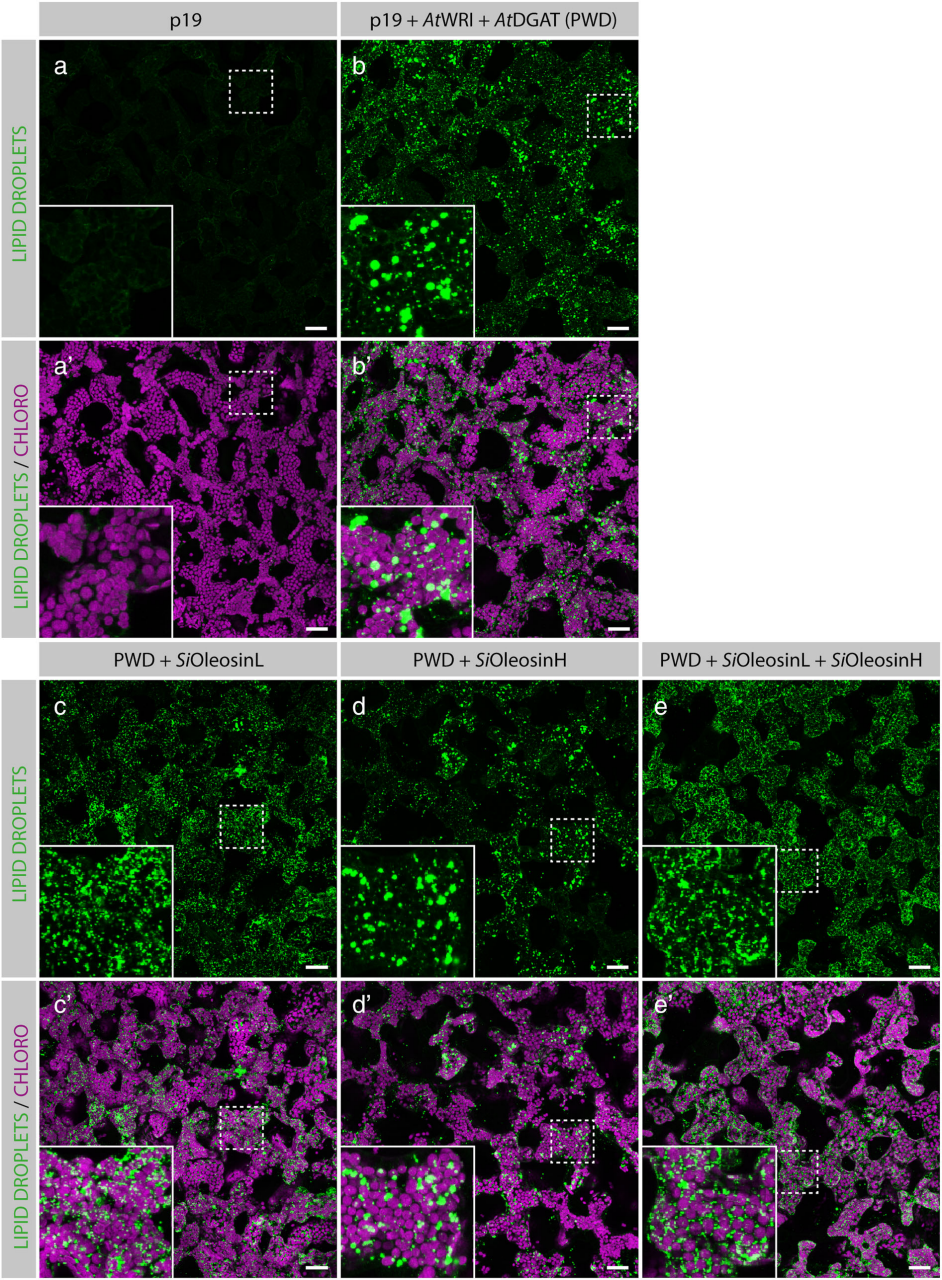
Visualization of lipid droplets in fresh *Nicotiana benthamiana* leaf transiently co‐expressing p19 (a and a′), and *At*WRI1 + *At*DGAT1 alone (b and b′) or in combination with *Si*OleosinL (c and c′), *Si*OleosinH (d and d′), and *Si*OleosinL + *Si*OleosinH (e and e′). The abaxial epidermis was peeled off, and the spongy mesophyll cells were imaged. Chloroplasts are shown in magenta, and lipid droplets were stained with the neutral lipid stain BODIPY505/515 and are shown in green. Each image is a maximum projection of several images taken along the *z*‐axis, at intervals of 2 μm. The insets are higher magnification of the areas highlighted by dashed boxes in the same panel. Scale bars: 40 μm

**FIGURE 4:**
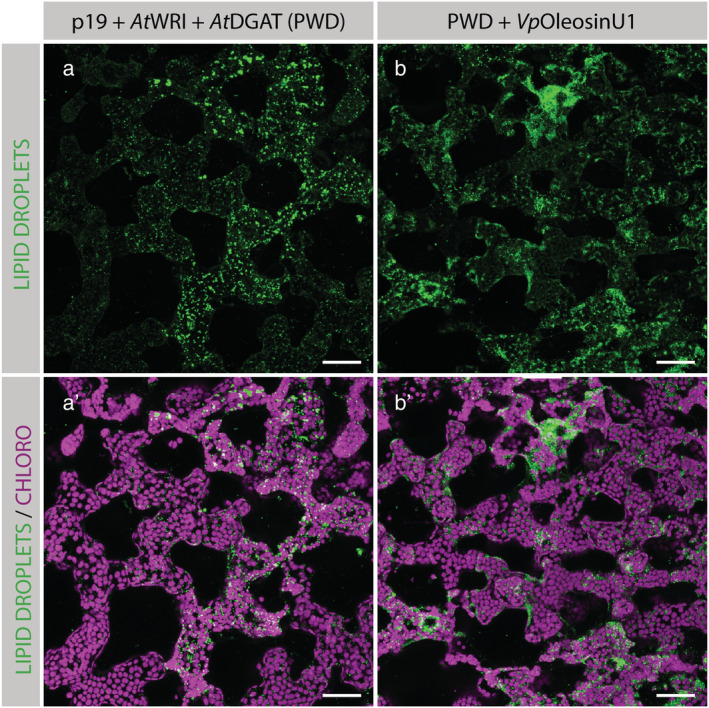
Visualization of lipid droplets in fresh *Nicotiana benthamiana* leaf transiently co‐expressing p19 + *At*WRI1 + *At*DGAT1 (PWD) alone (a and a′) or in combination with *Vp*OleosinU1 (b and b′). The abaxial epidermis was peeled off, and the spongy mesophyll cells were imaged. Chloroplasts are shown in magenta and lipid droplets were stained with the neutral lipid stain BODIPY505/515 and are shown in green. Each image is a maximum projection of several images taken along the *z* axis, at intervals of 2 μm. Scale bars: 50 μm

Next, we used confocal microscopy to test the effect of *Si*OleosinL and *Si*OleosinH on the size of accumulating lipid droplets in leaves. *Si*OleosinL expressed in a PWD background showed an accumulation of smaller lipid droplets (Figure 3c and c′) than in the PWD control (Figure 3b and b′). Using identical settings, LDs were barely detectable in the p19 control (Figure 3a and a′), confirming the results described in Figure 1. In contrast, the lipid droplets in leaves expressing PWD + *Si*OleosinH (Figure 3d and d′) were larger and looked similar to those observed in leaves expressing the PWD control. Finally, when *Si*OleosinH and *Si*OleosinL were co‐expressed with PWD (Figure 3e and e′), the lipid droplets were small in size and looked similar to those observed in leaves expressing PWD + *Si*OleosinL (Figure 3c and c′). In leaves expressing PWD + *Vp*OleosinU1 (Figure 4b and b′), the signal coming from lipid droplets appeared less punctate than in the PWD control leaves (Figure 4a and a′).

### 3.3 In vivo triacylglycerol storage stability

**FIGURE 5:**
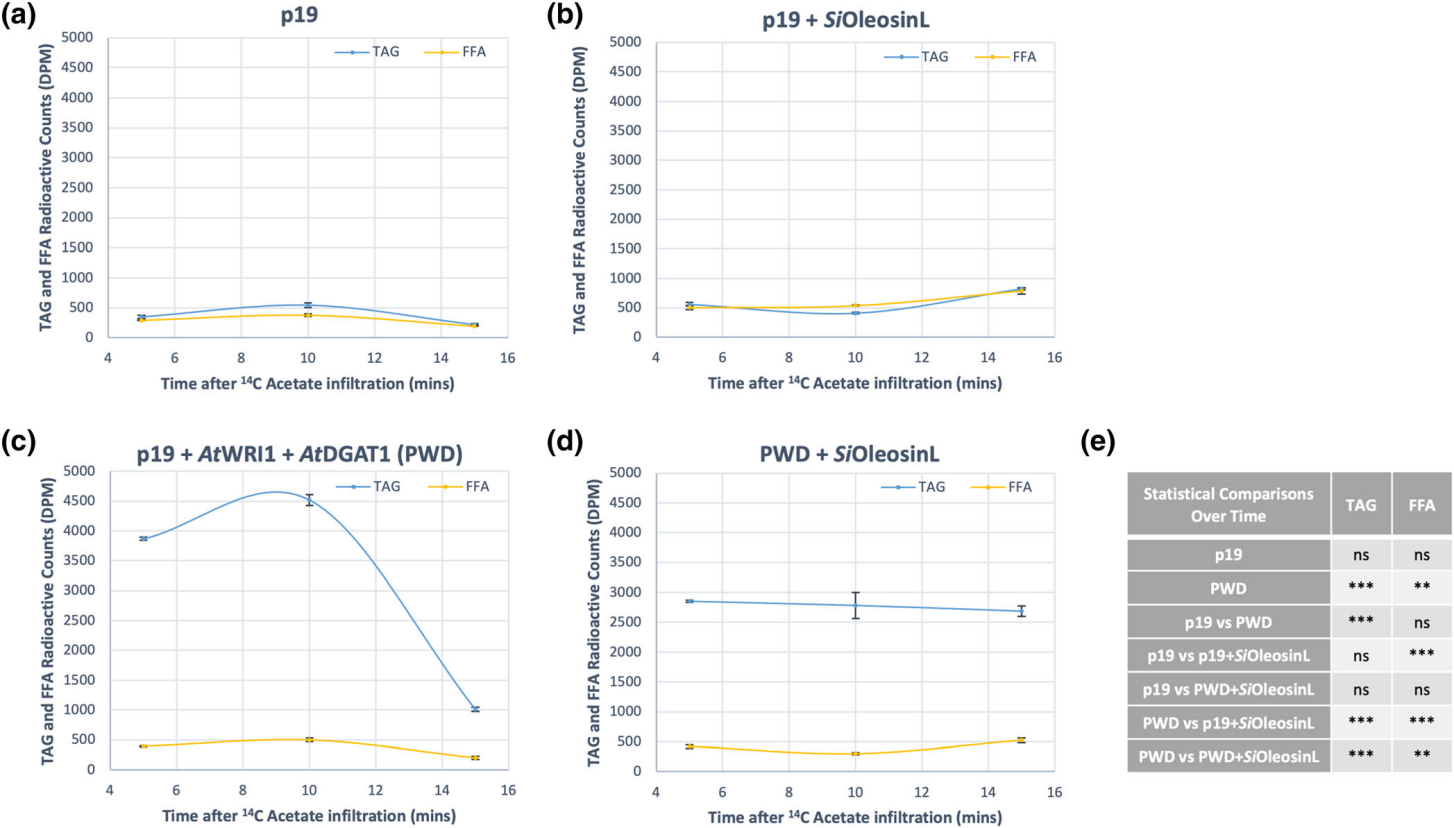
Triacylglycerol stability assay using [^14^C] acetate infiltration in *Nicotiana benthamiana* leaves transiently expressing (a) p19, (b) p19 + *Si*OleosinL, (c) p19 + *At*WRI1 + *At*DGAT1 (PWD), and (d) PWD + *Si*OleosinL, with (e) showing a summary of the statistical comparisons performed. Error bars (albeit small) denote standard error with *n* = 3. *** and **Significantly different at *p* < .001 and *p* < .01, respectively, against the p19 and PWD controls. ns indicates no statistical significance

To study the effect of LD proteins of interest on lipid accumulation, we conducted a [^14^C] acetate incorporation time course in *N. benthamiana* leaves. In the p19 only control, [^14^C] acetate incorporation into TAG did not exceed 500 disintegrations per minute (dpm) during the initial 15 min of feeding (Figure 5a). In contrast, *Si*OleosinL expression in the absence of *At*WRI1 and *At*DGAT1 increased TAG accumulation at 15 min (789 dpm, Figure 5b) compared with p19 (198 dpm, Figure 5a). In *N. benthamiana* leaves expressing p19 + *At*WRI1 + *At*DGAT1 (PWD) genes, TAG accumulated rapidly, reaching 3865 dpm at 5 min of [^14^C] acetate incorporation (Figure 5c) compared with 293 dpm in the p19 control (Figure 5a). This accumulation reached its maximum at 10 min after infiltration (4519 dpm). However, this spike in TAG accumulation quickly degraded to 1013 dpm at 15 min (Figure 5c). The additional co‐expression of *Si*OleosinL with *At*WRI1 and *At*DGAT1 (Figure 5d) showed that TAG rapidly accumulated at 5 min of feeding (2855 dpm) and remained constant at 10 and 15 min. At 15 min, TAG accumulation was equivalent to 2690 dpm in PWD + *Si*OleosinL (Figure 5d) compared with 1013 dpm in PWD alone (Figure 5c).

**FIGURE 6:**
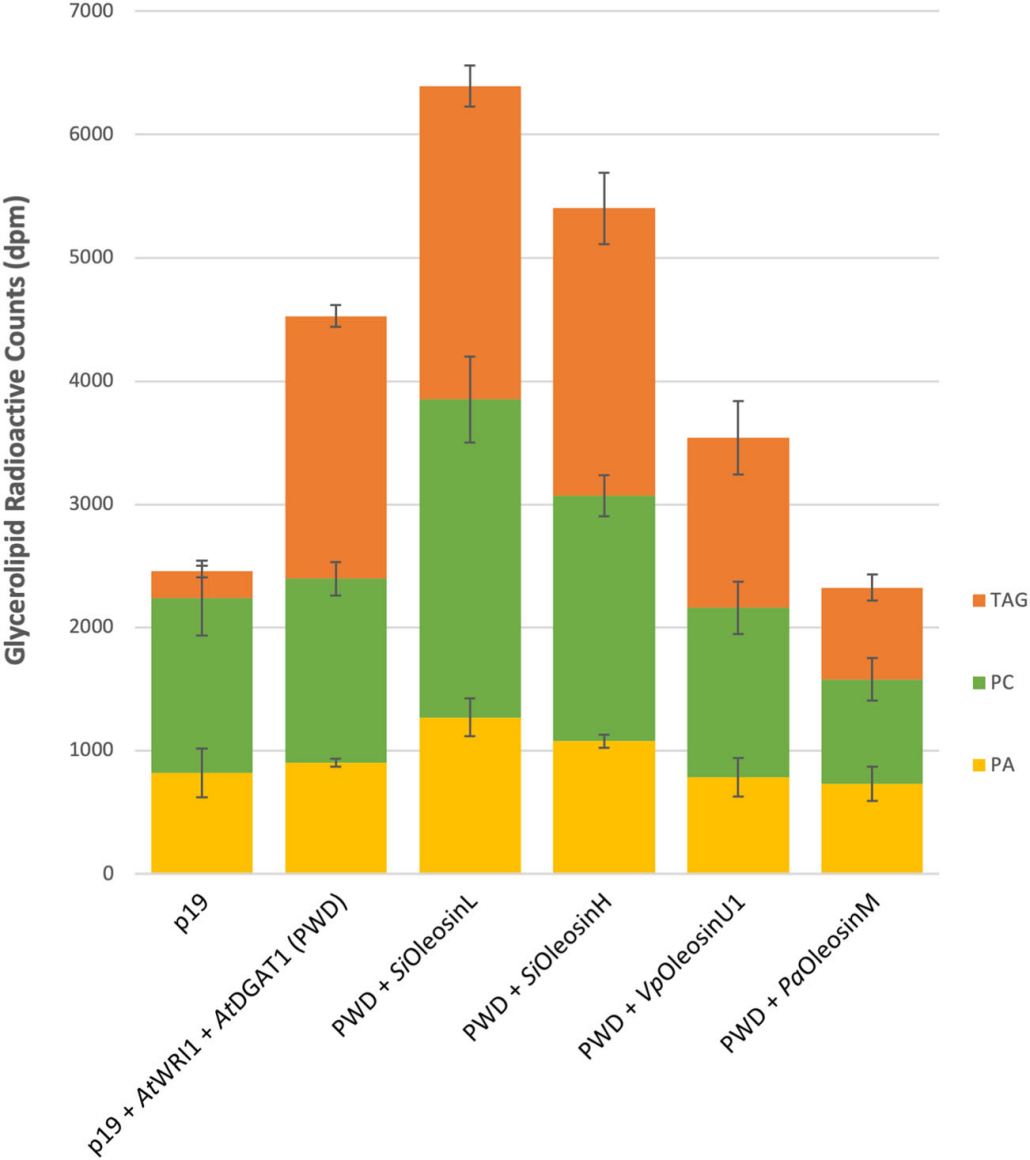
Radiolabeled triacylglycerol, phospholipid, and phosphatidic acid accumulation at 3 h of [^14^C] feeding in *Nicotiana benthamiana* leaves transiently expressing p19 + *At*WRI1 + *At*DGAT1 (PWD) in combination with *Si*OleosinL, *Si*OleosinH, vanilla leaf *Vp*OleosinU1, and avocado mesocarp *Pa*OleosinM. Error bars denote standard error with *n* = 3

In order to study degradation of the accumulated TAGs into its constituent derivatives, we analyzed [^14^C] acetate incorporation and TAG stability at 3 h post‐feeding (Figure 6). This assay showed a significant increase in phospholipids, mainly phosphatidylcholine (PC) (2579 dpm) and phosphatidic acid (PA) (1270 dpm) content in leaves expressing *Si*OleosinL compared with 1495 dpm of PC and 899 of PA in both p19 and PWD controls. In contrast, infiltrated leaves expressing *Si*OleosinH, vanilla leaf *Vp*OleosinU1 and avocado mesocarp *Pa*OleosinM did not show any significant effect on TAG, PC and PA contents compared with the p19 and PWD controls.

## 4 DISCUSSION

Earlier work focusing on the co‐expression of *At*WRI1 and *At*DGAT1 has illustrated a significant increase in the levels of TAG produced transiently in *N. benthamiana* leaf tissue (Vanhercke et al., 2013). However, only a few studies have expanded on this system by examining opportunities to better package newly synthesized TAGs and “protect” them from breakdown. In this study, we have investigated several oleosins for their potential to improve TAG packaging in non‐seed tissue using the existing *At*WRI1 and *At*DGAT1 system as a baseline for improvement.

### 4.1 *Si*OleosinL and *Si*OleosinH have different effects on TAG accumulation and lipid droplet size

The main finding of this study is that the co‐expression of *Si*OleosinL with *At*WRI1 and *At*DGAT1 genes significantly increased TAG content by 2.3‐fold compared with the expression of *At*WRI1 and *At*DGAT1 alone (Figure 1). This increase in TAG content was also correlated with a decrease in the size of accumulating lipid droplets (Figure 3c and c′). This suggests that *Si*OleosinL has a dual effect: it promotes TAG accumulation, and it reduces the size of the lipid droplets. In contrast, *Si*OleosinH co‐expressed with *At*WRI1 and *At*DGAT1 did not result in a significant increase in TAG content (Figure 1), nor did it reduce the size of the LDs produced transiently in *N. benthamiana* (Figure 3d and d′) compared with the PWD control (Figure 3b and b′). This demonstrates that both *S. indicum* oleosin isoforms affect LDs differently.

Interestingly, the co‐expression of both *Si*OleosinL and *Si*OleosinH together lead to a hybrid effect: TAGs did not accumulate significantly (Figure 1), but the LDs were reduced in size compared with those in *At*WRI1 and *At*DGAT1 controls (Figure 3b and b′). This suggests that the mechanisms controlling LD size and TAG accumulation are likely to be uncoupled, and that when *Si*OleosinL and *Si*OleosinH are co‐expressed, the L isoform has a dominant effect on size, whereas the H isoform has a dominant effect on TAG accumulation. These results suggest that *Si*OleosinL and *Si*OleosinH may regulate LD stability and degradation differently.

### 4.2 *Si*OleosinH may be affected by ubiquitination

Indeed, some oleosin isoforms contain C‐terminal domain ubiquitination sites, which allow for the regulation of LDs via ubiquitin‐dependent degradation pathways (Hsiao & Tzen, 2011; Tai et al., 2002). Ubiquitin‐dependent degradation of oleosins or LDs containing oleosins has previously been reported in sesame seedlings (Hsiao & Tzen, 2011). Ubiquitination is a post‐translational modification in which ubiquitin chains or single ubiquitin molecules are appended to target proteins, thereby affecting protein longevity, as well as protein activity and/or localization (Guerra & Callis, 2012). More specifically, ubiquitination was shown to control the fate of oleosins, as well as regulate lipid droplet dynamics in plants (Deruyffelaere et al., 2015).

Interestingly, *Si*OleosinH has three predicted ubiquitination sites at residues 130, 143, and 145 (Figure S1), whereas *Si*OleosinL lacks any ubiquitination sites (Hsiao & Tzen, 2011). This observation is compatible with the results we have presented in this study, and we hypothesize that the ubiquitination sites on *Si*OleosinH may counteract the positive stabilizing effects that *Si*OleosinL imparts on TAG accumulation in *N. benthamiana* leaf tissue. Although protein ubiquitination is integral to many biological pathways, such as proteasomal degradation, stress responses, hormone biosynthesis and signaling, morphogenesis, and battling pathogens (Sorokin et al., 2009), LD instability is not desired when trying to increase lipid accumulation in vegetative tissue. Although beyond the scope of this study, it will be interesting to further investigate the functions of the SiOleosinH ubiquitination sites and the effects that variably ubiquitinated *Si*OleosinH mutants may have on TAG accumulation. The limited amount of oil produced in leaves transiently expressing these constructs suggests that these experiments would be better suited to testing in stable transformants.

### 4.3 TAG accumulation time course and composition

Radiolabeled assays were used in this study to investigate the storage stability of the TAGs accumulated in vivo, as well as to analyze the lipid content present in the LDs produced transiently in *N. benthamiana*. These assays showed that although there was initially a high amount of TAG present in leaves expressing *At*WRI1 and *At*DGAT1 (Figure 5c), it was rapidly degraded after 15 min. Similarly, there was no significant TAG accumulation observed when *Si*OleosinL was expressed alone (Figure 5b). However, when *At*WRI1, *At*DGAT1 and *Si*OleosinL were all co‐expressed, there was no rapid degradation of radiolabeled TAG observed in these leaves (Figure 5d), albeit the initial amount of TAG present at 5 min was moderate compared with the expression of *At*WRI1 and *At*DGAT1 alone. This suggests that the co‐expression of *Si*OleosinL in the *At*WRI1 and *At*DGAT1 background was able to prevent TAG degradation, demonstrating the oil packaging stability provided by this LD protein.

Further analyses at 3 h post‐feeding indicate degradation products that mainly consisted of PC and PA in the leaves expressing *Si*OleosinL (Figure 6). Interestingly, the amount of TAG present in these leaves remained largely unchanged over the course of the 3 h assay. As the amount of [^14^C] acetate used was in excess, the constant amount of TAGs was most likely attributed to the stability of TAG storage imparted by *Si*OleosinL, rather than to a limitation in the amount of [^14^C] acetate infiltrated. Additionally, the constant amount of TAGs in the leaves co‐expressing *At*WRI1, *At*DGAT1 and *Si*OleosinL (Figure 5d), coupled with the rapid TAG degradation observed in the absence of *Si*OleosinL (Figure 5c) suggests that the lipid synthesis‐to‐degradation ratio is likely constant in the infiltrated *N. benthamiana* leaves expressing *Si*OleosinL.

### 4.4 Effect of other oleosins on LDs

Another oleosin with interesting characteristics was *Vp*OleosinU1. Here, vanilla leaf oleosin showed an increase in LD abundance, which was distributed in a different pattern compared with *Si*OleosinL (Figures 3 and 4). This is in coherence with published data on vanilla LD characteristics, where the epidermis of vanilla leaves and most other Asparagales species are known to have clustered LDs (< .5 μm), which is speculated to be needed for cuticle formation (Huang & Huang, 2016).

## 5 CONCLUSION

In summary, each oleosin tested in this study showed a differentiated effect on several aspects of LD production and protection, such as increasing total oil content, modifying oil composition and regulating lipid droplet size. We have demonstrated that out of the Oleosins tested in this study, *Si*OleosinL was best able to package oils produced by the overexpression of *At*WRI1 and *At*DGAT1 in leaf tissue. This new knowledge can inform future metabolic engineering approaches to meet increasing oil demands through high biomass plant genetics.

## CONFLICT OF INTERESTS

The authors declare that the research was conducted in the absence of any commercial or financial relationships that could be construed as a potential conflict of interest.

## AUTHOR CONTRIBUTIONS

S.Y. carried out the lipid experiments, conducted statistical analyses, and contributed to drafting and finalizing the manuscript. V.R. performed microscopy experiments, analyzed results, and participated in drafting and finalizing the manuscript. K.R. contributed to radiolabeled assays, experiments, and analyses of data, and participated in manuscript review. P.S. contributed to radiolabeled assays. T.V. designed constructs and participated in discussions and manuscript review. L.M. assisted with lipid analysis. S.S. conceptualized the study and participated in manuscript review. J.P. conceptualized the study. A.E.T. supervised the study and contributed to drafting and finalizing the manuscript. S.Y. and V.R. agree to serve as the authors responsible for contact and ongoing communication about this work.

## NOMENCLATURE

dpi: days post infiltration
dpm: disintegrations per minute
DW: dry weight
FA: fatty acid
FFA: free fatty acid
LD: lipid droplet
PA: phosphatidic acid
PC: phosphatidylcholine
PWD: p19 + AtWRI1 + AtDGAT1
TAG: triacylglycerol

## Supporting information

**Figure S1.** Sequence alignment of *Sesamum indicum* oleosin L and H protein isoforms. *Si*OleosinH ubiquitination sites are highlighted in yellow (residue 130, 143, and 145).
