# Supplementary material for: Sesamum indicum Oleosin L improves oil packaging in Nicotiana benthamiana leaves
Source: Plant Direct. 2021 Sep 6;5(9):e343. doi: 10.1002/pld3.343 (PMC8421512; doi:10.1002/pld3.343)
Supplement: Supplementary file 1 — Figure S1. Sequence alignment of Sesamum indicum oleosin L and H protein isoforms. SiOleosinH ubiquitination sites are highlighted in yellow (residue 130, 143, and 145). [file PLD3-5-e343-s001.pdf]

(from 1-1070 bp)

pOIL380: Nb-optimized RoTadA in pJP3343 (7884 bp)

TCCTGTGGTTGGCATGCACATACAAATGGACGAACGGATAAACCTTTTCACGCCCTTTTAAATATCCGATTATTCTAATAAACGCTCTTTTCTCTTAGGTTTACCCG

T-DNA right border

20 40 60 80 100

CCAATATATCCTGTCAAACACTGATAGTTTAAACTGAAGGCGGGAAACGACAATCTGCTAGTGGATCTCCCAGTCACGACGTTGTAAAACGGGCGCCCCGCGGAAAG

T-DNA right border

120 140 160 180 200

CTTGCTAGCCAATTGGGGCCCAACGTTCTCGAGTTTTTCTAGAAGGCCTTGGATCCCATGGAGTCAAAGATTCAAATAGAGGACCTAACAGAACTCGCCGTAAAGAC

Pro_35Sx2

220 240 260 280 300 320

TGGCGAACAGTTCATACAGAGTCTCTTACGACTCAATGACAAGAAGAAAATCTTCGTCAACATGGTGGAGCACGACACACTTGTCTACTCCAAAAATATCAAAGATA

Pro_35Sx2

340 360 380 400 420

CAGTCTCAGAAGACCAAAGGGCAATTGAGACTTTTCAACAAAGGGTAATATCCGGAAACCTCCTCGGATTCCATTGCCCAGCTATCTGTCACTTTATTGTGAAGATA

Pro_35Sx2

440 460 480 500 520

GTGGAAAAGGAAGGTGGCTCCTACAAATGCCATCATTGCGATAAAGGAAAGGCCATCGTTGAAGATGCCTCTGCCGACAGTGGTCCCAAAGATGGACCCCCACCCAC

Pro_35Sx2

540 560 580 600 620 640

GAGGAGCATCGTGGAAAAAGAAGACGTTCCAACCACGTCTTCAAAGCAAGTGGATTGATGTGATATCTCCACTGACGTAAGGGATGACGCACAATCCCACTATCCTT

Pro_35Sx2

660 680 700 720 740

CGCAAGACCCTTCCTCTATATAAGGAAGTTCATTTCATTTGGAGAGAACACGGGGGACTGAATTCGTCGACTTTGCGGCCGCATGACTGATCAAAAGACCATCGATA

Pro_35Sx2 RoTadA (opt)

760 780 800 820 840

GCGTTAAGACCAGCCTGTACGCTGCTGTTGGTGCTGGTGATGTTGTTGTTCAGGCTGTTGCTGATGTTGTGGCTCAGGTTAGGTCTAGGGCTGAATCTACTCAGGGT

RoTadA (opt)

860 880 900 920 940 960

GATGTGGAAGAAAGGGTGGGAGGTGCTAAAGAGAGGATTGCTGGACTTCAAGAAGAGGTGACAGAGGGAGTTGAGAACCTTAGGGATAGGCTTGCTGGTCTGCCTTC

RoTadA (opt)

980 1,000 1,020 1,040 1,060

pOIL380: Nb-optimized RoTadA in pJP3343 (7884 bp) (from 1071-2140 bp)

TGAACTTCCTGAAGAGTTGGCTGAGCTGAGGGAAAAGTTCACCGCTGATGAGCTTAGGAAGGTGGCAGAGGCTTACCTGAAGGTTGCAAGCGATCTTTACACCTCTC

RoTadA (opt)

1,080 1,100 1,120 1,140 1,160

TTGCTGAGAGGGGTGAGGATACCGTTGAGAGAATTAGGAAGCAGCCTGTTGTTGAAGAGGGTATCGGTAGAGCTGAGACTGCTTTCGGTGATGCTGTTGAGCTTACC

RoTadA (opt)

1,180 1,200 1,220 1,240 1,260 1,280

GAAGAGGCTCTTGGTACTGTTGCTAGACAGACTAGAGCTGTTGGAGAGCAGGCTGCTAAGTTGGCTGGTAGAGCTTCTGGTAGGATCTCTGATACTGCTGAGGGTCT

RoTadA (opt)

1,300 1,320 1,340 1,360 1,380

TGGTGAGGCTATTGCAGATGCTGGTGATGAGGCTGCTCTTAAGGTGCTGGATCTTGGTGATCAAGCTGAAGAGGCTAGCAAGGATGCTGCTGATAGAGTTGCTGCTA

RoTadA (opt)

1,400 1,420 1,440 1,460 1,480

CAGCTGCTGATGTGCAAGCTCAAGCTGATAAGGCTGCACAAGCTAAGCACGCTGCTCCAGCTAAAAAGGCTGCTCCTGCAAAAGCTGCTGCTACTCCAGCTCCAGCA

RoTadA (opt)

1,500 1,520 1,540 1,560 1,580 1,600

CCAGCTAAGAAAGTTGCTGCACCAGCAAAAAAAGCTGCTCCAGCTAAGAAAGCTTAGACTAGTGATATCCCTGTGTGAAATTGTTATCCGCTAGCGTGATCGTTCAA

RoTadA (opt) NO...r

1,620 1,640 1,660 1,680 1,700

ACATTTGGCAATAAAGTTTCTTAAGATTGAATCCTGTTGCCGGTCTTGCGATGATTATCATATAATTTCTGTTGAATTACGTTAAGCATGTAATAATTAACATGTAA

NOS terminator

1,720 1,740 1,760 1,780 1,800

TGCATGACGTTATTTATGAGATGGGTTTTTATGATTAGAGTCCCGCAATTATACATTTAATACGCGATAGAAAACAAAATATAGCGCGCAAACTAGGATAAATTATC

NOS terminator

1,820 1,840 1,860 1,880 1,900 1,920

GCGCGCGGTGTCATCTATGTTACTAGATCCCATGGGAAGTTCCTATTCCGAAGTTCCTATTCTCTGAAAAGTATAGGAACTTCAGCGATCGCAGACGTCGGGATCTT

NOS terminator FRT site

1,940 1,960 1,980 2,000 2,020

CTGCAAGCATCTCTATTTCCTGAAGGTCTAACCTCGAAGATTTAAGATTTAATTACGTTTATAATTACAAAATTGATTCTAGTATCTTTAATTTAATGCTTATACAT

enTCUP2 promoter

2,040 2,060 2,080 2,100 2,120 2,140

pOIL380: Nb-optimized RoTadA in pJP3343 (7884 bp) (from 2141-3210 bp)

TATTAATTAATTTAGTACTTTCAATTTGTTTTCAGAAATTATTTTACTATTTTTTATAAAATAAAAGGGAGAAAATGGCTATTTAAATACTAGCCTATTTTATTTCA

enTCUP2 promoter

2,160 2,180 2,200 2,220 2,240

ATTTTAGCTTAAAATCAGCCCCAATTAGCCCCAATTTCAAATTCAAATGGTCCAGCCCAATTCCTAAATAACCCACCCCTAACCCGCCCGGTTTCCCCTTTTGATCC

enTCUP2 promoter

2,260 2,280 2,300 2,320 2,340

ATGCAGTCAACGCCCAGAATTTCCCTATATAATTTTTTAATTCCCAAACACCCCTAACTCTATCCCATTTCTCACCAACCGCCACATAGATCTATCCTCTTATCTCT

enTCUP2 promoter

2,360 2,380 2,400 2,420 2,440 2,460

CAAACTCTCTCGAACCTTCCCCTAACCCTAGCAGCCTCTCATCATCCTCACCTCAAAACCCACCGGGGCCGGCCATGATTGAACAAGATGGATTGCACGCAGGTTCT

enTCUP2 promoter | neomycin phos...ansferase II

2,480 2,500 2,520 2,540 2,560

CCGGCCGCTTGGGTGGAGAGGCTATTCGGCTATGACTGGGCACAACAGACAATCGGCTGCTCTGATGCCGCCGTGTTCCGGCTGTCAGCGCAGGGGAGGCCGGTTCT

neomycin phosphotransferase II

2,580 2,600 2,620 2,640 2,660

TTTTGTCAAGACCGACCTGTCCGGTGCCCTGAATGAACTTCAAGACGAGGCAGCGCGGCTATCGTGGCTGGCCACGACGGGCGTTCCTTGCGCAGCTGTGCTCGACG

neomycin phosphotransferase II

2,680 2,700 2,720 2,740 2,760 2,780

TTGTCACTGAAGCGGGAAGGGACTGGCTGCTATTGGGCGAAGTGCCGGGGCAGGATCTCCTGTCATCTCACCTTGCTCCTGCCGAGAAAGTATCCATCATGGCTGAT

neomycin phosphotransferase II

2,800 2,820 2,840 2,860 2,880

GCAATGCGGCGGCTGCATACGCTTGATCCGGCTACCTGCCCATTCGACCACCAAGCGAAACATCGCATCGAGCGAGCACGTACTCGGATGGAAGCCGGTCTTGTCGA

neomycin phosphotransferase II

2,900 2,920 2,940 2,960 2,980

TCAGGATGATCTGGACGAAGAGCATCAGGGGCTCGCGCCAGCCGAACTGTTCGCCAGGCTCAAGGCGCGCATGCCCGACGGCGAGGATCTCGTCGTGACTCATGGCG

neomycin phosphotransferase II

3,000 3,020 3,040 3,060 3,080 3,100

ATGCCTGCTTGCCGAATATCATGGTGGAAAATGGCCGCTTTTCTGGATTCATCGACTGTGGCCGGCTGGGTGTGGCGGACCGCTATCAGGACATAGCGTTGGCTACC

neomycin phosphotransferase II

3,120 3,140 3,160 3,180 3,200

pOIL380: Nb-optimized RoTadA in pJP3343 (7884 bp) (from 3211-4280 bp)

CGTGATATTGCTGAAGAGCTTGGCGGCGAATGGGCTGACCGCTTCCTCGTGCTTTACGGTATCGCCGCTCCCGATTCGCAGCGCATCGCCTTCTATCGCCTTCTTGA
neomycin phosphotransferase II
3,220 3,240 3,260 3,280 3,300

CGAGTTCTTCTGAGGCGCGCCGATCGTTCAAACATTTGGCAATAAAGTTTCTTAAGATTGAATCCTGTTGCCGGTCTTGCGATGATTATCATATAATTTCTGTTGAA
neo...II NOS terminator
3,320 3,340 3,360 3,380 3,400 3,420

TTACGTTAAGCATGTAATAATTAACATGTAATGCATGACGTTATTTATGAGATGGGTTTTTATGATTAGAGTCCCGCAATTATACATTTAATACGCGATAGAAAACA
NOS terminator
3,440 3,460 3,480 3,500 3,520

AAATATAGCGCGCAAACTAGGATAAATTATCGCGCGCGGTGTCATCTATGTTACTAGATCCCTAGGGAAGTTCCTATTCCGAAGTTCCTATTCTCTGAAAAGTATAG
NOS terminator FRT site
3,540 3,560 3,580 3,600 3,620

GAACTTCTTTGCGTATTGGGCGCTCTTGGCCTTTTTGGCCACCGGTCGTACGGTTAAAACCACCCCAGTACATTAAAAACGTCCGCAATGTGTTATTAAGTTGTCTA
T-DNA left border
3,640 3,660 3,680 3,700 3,720 3,740

AGCGTCAATTTGTTTACACCACAATATATCCTGCCACCAGCCAGCCAACAGCTCCCCGACCGGCAGCTCGGCACAAAATCACCACTCGATACAGGCAGCCCATCAGT
T-DNA left border
3,760 3,780 3,800 3,820 3,840

CCACTAGACGCTCACCGGGCTGGTTGCCCTCGCCGCTGGGCTGGCGGCCGTCTATGGCCCTGCAAACGCGCCAGAAACGCCGTCGAAGCCGTGTGCGAGACACCGCA
RK2 oriV
3,860 3,880 3,900 3,920 3,940

GCCGCCGGCGTTGTGGATACCTCGCGGAAAACTTGGCCCTCACTGACAGATGAGGGGCGGACGTTGACACTTGAGGGGCCGACTCACCCGGCGCGGCGTTGACAGAT
RK2 oriV
3,960 3,980 4,000 4,020 4,040 4,060

GAGGGGCAGGCTCGATTTCGGCCGGCGACGTGGAGCTGGCCAGCCTCGCAAATCGGCGAAAACGCCTGATTTTACGCGAGTTTCCCACAGATGATGTGGACAAGCCT
RK2 oriV
4,080 4,100 4,120 4,140 4,160

GGGGATAAGTGCCCTGCGGTATTGACACTTGAGGGGCGCGACTACTGACAGATGAGGGGCGCGATCCTTGACACTTGAGGGGCAGAGTGCTGACAGATGAGGGGCGC
RK2 oriV
4,180 4,200 4,220 4,240 4,260 4,280

pOIL380: Nb-optimized RoTadA in pJP3343 (7884 bp) (from 4281-5350 bp)

ACCTATTGACATTTGAGGGGCTGTCCACAGGCAGAAAATCCAGCATTTGCAAGGGTTTCCGCCCGTTTTTCGGCCACCGCTAACCTGTCTTTTAACCTGCTTTTAAA

RK2 oriV

4,300 4,320 4,340 4,360 4,380

CCAATATTTATAAACCTTGTTTTTAACCAGGGCTGCGCCCTGTGCGCGTGACCGCGCACGCCGAAGGGGGGTGCCCCCCCTTCTCGAACCCTCCCGGCCCGCTCTCG

RK2 oriV

4,400 4,420 4,440 4,460 4,480

CGTTGGCAGCATCACCCATAATTGTGGTTTCAAAATCGGCTCCGTCGATACTATGTTATACGCCAACTTTGAAAACAACTTTGAAAAAGCTGTTTTCTGGTATTTAA

4,500 4,520 4,540 4,560 4,580 4,600

GGTTTTTAGAATGCAAGGAACAGTGAATTGGAGTTCGTCTTGTTATAATTAGCTTCTTGGGGTATTTAAATACTGTAGAAAAGAGGAAGGAAATAATAAATGGCTAAA

4,620 4,640 4,660 4,680 4,700

ATGAGAATATCACCGGAATTGAAAAAACTGATCGAAAAATACCGCTGCGTAAAAGATACGGAAGGAATGTCTCCTGCTAAGGTATATAAGCTGGTGGGAGAAAATGA

neomycin-kanamycin phosphotransferase type III

4,720 4,740 4,760 4,780 4,800

AAACCTATATTTAAAAATGACGGACAGCCGGTATAAAGGGACCACCTATGATGTGGAACGGGAAAAGGACATGATGCTATGGCTGGAAGGAAAGCTGCCTGTTCCAA

neomycin-kanamycin phosphotransferase type III

4,820 4,840 4,860 4,880 4,900 4,920

AGGTCCTGCACCTTGAACGGCATGATGGCTGGAGCAATCTGCTCATGAGTGAGGCCGATGGCGTCCTTTGCTCGGAAGAGTATGAAGATGAACAAAGCCCTGAAAAG

neomycin-kanamycin phosphotransferase type III

4,940 4,960 4,980 5,000 5,020

ATTATCGAGCTGTATGCGGAGTGCATCAGGCTCTTTCACTCCATCGACATATCGGATTGTCCCTATACGAATAGCTTAGACAGCCGCTTAGCCGAATTGGATTACTT

neomycin-kanamycin phosphotransferase type III

5,040 5,060 5,080 5,100 5,120

ACTGAATAACGATCTGGCCGATGTGGATTGCGAAAACTGGGAAGAAGACACCCCATTTAAAGATCCGCGCGAGCTGTATGATTTTTTAAAGACGGAAAAGCCCGAAG

neomycin-kanamycin phosphotransferase type III

5,140 5,160 5,180 5,200 5,220 5,240

AGGAACTTGTCTTTTCCCACGGCGACCTGGGAGACAGCAACATCTTTGTGAAAGATGGCAAAGTAAGTGGCTTTATTGATCTTGGGAGAAGCGGCAGGGCGGACAAG

neomycin-kanamycin phosphotransferase type III

5,260 5,280 5,300 5,320 5,340

pOIL380: Nb-optimized RoTadA in pJP3343 (7884 bp) (from 5351-6527 bp)

TGGTATGACATTGCCTTCTGCGTCCGGTCGATCAGGGAGGATATTGGGGAAGAACAGTATGTCGAGCTATTTTTTGACTTACTGGGGATCAAGCCTGATTGGGAGAA

neomycin-kanamycin phosphotransferase type III

5,360 5,380 5,400 5,420 5,440

AATAAAATATTATATTTTACTGGATGAATTGTTTTAGTACCTAGATGTGGCGCAACGATGCTGGCGACAAGCAGGAGCGCACCGACTTCTTCCGCATCAAGTGTTTT

neomycin-kanamy...erase type III

5,460 5,480 5,500 5,520 5,540 5,560

GGCTCTCAGGCCGAGGCCCACGGCAAGTATTTGGGCAAGGGGTCGCTGGTATTCGTGCAGGGCAAGATTCGGAATACCAAGTACGAGAAGGACGGCCAGACGGTCTA

5,580 5,600 5,620 5,640 5,660

CGGGACCGACTTCATTGCCGATAAGGTGGATTATCTGGACACCAAGGCACCAGGCGGATCAAATCAGGAATAAGGGCACATTGCCCCGGCGTGAGTCGGGGCAATCC

5,680 5,700 5,720 5,740 5,760

CGCAAGGAGGGTGAATGAATCGGACGTTTGACCGGAAGGCATACAGGCAAGAACTGATCGACGCGGGGTTTTCCGCCGAGGATGCCGAAACCATCGCAAGCCGCACC

derived from plasmid RK2

5,780 5,800 5,820 5,840 5,860 5,880

GTCATGCGTGCGCCCCGCGAAACCTTCCAGTCCGTCGGCTCGATGGCCCAGCAAGCTACGGCCAAGATCGAGCGCGACAGCGTGCAACTGGCTCCCCCTGCCCTGCC

derived from plasmid RK2

5,900 5,920 5,940 5,960 5,980

CGCGCCATCGGCCGCCGTGGAGCGTTCGCGTCGTCTCGAACAGGAGGCGGCAGGTTTGGCGAAGTCGATGACCATCGACACGCGAGGAACTATGACGACCAAGAAGC

derived from plasmid RK2

6,000 6,020 6,040 6,060 6,080

GAAAAACCGCCGGCGAGGACCTGGCAAAACAGGTCAGCGAGGCCAAGCAAGCCGCGTTGCTGAAACACACGAAGCAGCAGATCAAGGAAATGCAGCTTTCCTTGTTC

derived from plasmid RK2

6,100 6,120 6,140 6,160 6,180 6,200

GATATTGCGCCGTGGCCGGACACGATGCGAGCGATGCCAAACGACACGGCCCGCTCTGCCCTGTTCACCACGCGCAACAAGAAAATCCCGCGCGAGGCGCTGCAAAA

derived from plasmid RK2

6,220 6,240 6,260 6,280 6,300

CAAGGTCATTTTCCACGTCAACAAGGACGTGAAGATCACCTACACCGGCGTCGAGCTGCGGGCCGACGATGACGAACTGGTGTGGCAGCAGGTGTTGGAGTACGCGA

derived from plasmid RK2

6,320 6,340 6,360 6,380 6,400 6,420

AGCGCACCCCTATCGGCGAGCCGATCACCTTCACGTTCTACGAGCTTTGCCAGGACCTGGGCTGGTCGATCAATGGCCGGTATTACACGAAGGCCGAGGAATGCCTG

derived from plasmid RK2

6,440 6,460 6,480 6,500 6,520

pOIL380: Nb-optimized RoTadA in pJP3343 (7884 bp) (from 6528-7597 bp)

TCGCGCCTACAGGCGACGGCGATGGGCTTCACGTCCGACCGCGTTGGGCACCTGGAATCGGTGTCGCTGCTGCACCGCTTCCGCGTCCTGGACCGTGGCAAGAAAAC

derived from plasmid RK2

6,540 6,560 6,580 6,600 6,620

GTCCCGTTGCCAGGTCCTGATCGACGAGGAAATCGTCGTGCTGTTTGCTGGCGACCACTACACGAAATTCATATGGGAGAAGTACCGCAAGCTGTCGCCGACGGCCC

derived from plasmid RK2

6,640 6,660 6,680 6,700 6,720 6,740

GACGGATGTTCGACTATTTCAGCTCGCACCGGGAGCCGTACCCGCTCAAGCTGGAAACCTTCCGCCTCATGTGCGGATCGGATTCCACCCGCGTGAAGAAGTGGCGC

derived from plasmid RK2

6,760 6,780 6,800 6,820 6,840

GAGCAGGTCGGCGAAGCCTGCGAAGAGTTGCGAGGCAGCGGCCTGGTGGAACACGCCTGGGTCAATGATGACCTGGTGCATTGCAAACGCTAGGGCCTTGTGGGGTC

derived from plasmid RK2

6,860 6,880 6,900 6,920 6,940

AGTTCCGGCTGGGGGTTCAGCAGCCAGCGCTTTACTGAGATCCTCTTCCGCTTCCTCGCTCACTGACTCGCTGCGCTCGGTCGTTCGGCTGCGGCGAGCGGTATCAG

ColE1

6,960 6,980 7,000 7,020 7,040 7,060

CTCACTCAAAGGCGGTAATACGGTTATCCACAGAATCAGGGGATAACGCAGGAAAGAACATGTGAGCAAAAGGCCAGCAAAAGGCCAGGAACCGTAAAAAGGCCGCG

ColE1

7,080 7,100 7,120 7,140 7,160

TTGCTGGCGTTTTTCCATAGGCTCCGCCCCCCTGACGAGCATCACAAAAATCGACGCTCAAGTCAGAGGTGGCGAAACCCGACAGGACTATAAAGATACCAGGCGTT

ColE1

7,180 7,200 7,220 7,240 7,260

TCCCCCTGGAAGCTCCCTCGTGCGCTCTCCTGTTCCGACCCTGCCGCTTACCGGATACCTGTCCGCCTTTCTCCCTTCGGGAAGCGTGGCGCTTTCTCATAGCTCAC

ColE1

7,280 7,300 7,320 7,340 7,360 7,380

GCTGTAGGTATCTCAGTTCGGTGTAGGTCGTTCGCTCCAAGCTGGGCTGTGTGCACGAACCCCCCGTTCAGCCCGACCGCTGCGCCTTATCCGGTAACTATCGTCTT

ColE1

7,400 7,420 7,440 7,460 7,480

GAGTCCAACCCGGTAAGACACGACTTATCGCCACTGGCAGCAGCCACTGGTAACAGGATTAGCAGAGCGAGGTATGTAGGCGGTGCTACAGAGTTCTTGAAGTGGTG

ColE1

7,500 7,520 7,540 7,560 7,580

pOIL380: Nb-optimized RoTadA in pJP3343 (7884 bp) (from 7598-7884 bp)

GCCTAACTACGGCTACACTAGAAGAACAGTATTTGGTATCTGCGCTCTGCTGAAGCCAGTTACCTTCGGAAAAAGAGTTGGTAGCTCTTGATCCGGCAAACAAACCA

ColE1

7,600 7,620 7,640 7,660 7,680 7,700

CCGCTGGTAGCGGTGGTTTTTTTGTTTGCAAGCAGCAGATTACGCGCAGAAAAAAAGGATCTCAAGAAGATCCTTTGATCTTTTCTACGGGGTCTGACGCTCAGTGG

ColE1

7,720 7,740 7,760 7,780 7,800

AACGAAAACTCACGTTAAGGGATTTTGGTCATGAGATTATCAAAAAGGATCTTCACCTAGATCCTTTTGGATC

ColE1

7,820 7,830 7,840 7,850 7,860 7,870 7,880

(from 1-1070 bp)

pOIL382: Nb-optimized SiOleosinL in pJP3343

TCCTGTGGTTGGCATGCACATACAAATGGACGAACGGATAAACCTTTTCACGCCCTTTTAAATATCCGATTATTCTAATAAACGCTCTTTTCTCTTAGGTTTACCCG

T-DNA right border

20 40 60 80 100

CCAATATATCCTGTCAAACACTGATAGTTTAAACTGAAGGCGGGAAACGACAATCTGCTAGTGGATCTCCCAGTCACGACGTTGTAAAACGGGCGCCCCGCGGAAAG

T-DNA right border

120 140 160 180 200

CTTGCTAGCCAATTGGGGCCCAACGTTCTCGAGTTTTTCTAGAAGGCCTTGGATCCCATGGAGTCAAAGATTCAAATAGAGGACCTAACAGAACTCGCCGTAAAGAC

Pro_35Sx2

220 240 260 280 300 320

TGGCGAACAGTTCATACAGAGTCTCTTACGACTCAATGACAAGAAGAAAATCTTCGTCAACATGGTGGAGCACGACACACTTGTCTACTCCAAAAATATCAAAGATA

Pro_35Sx2

340 360 380 400 420

CAGTCTCAGAAGACCAAAGGGCAATTGAGACTTTTCAACAAAGGGTAATATCCGGAAACCTCCTCGGATTCCATTGCCCAGCTATCTGTCACTTTATTGTGAAGATA

Pro_35Sx2

440 460 480 500 520

GTGGAAAAGGAAGGTGGCTCCTACAAATGCCATCATTGCGATAAAGGAAAGGCCATCGTTGAAGATGCCTCTGCCGACAGTGGTCCCAAAGATGGACCCCCACCCAC

Pro_35Sx2

540 560 580 600 620 640

GAGGAGCATCGTGGAAAAAGAAGACGTTCCAACCACGTCTTCAAAGCAAGTGGATTGATGTGATATCTCCACTGACGTAAGGGATGACGCACAATCCCACTATCCTT

Pro_35Sx2

660 680 700 720 740

CGCAAGACCCTTCCTCTATATAAGGAAGTTCATTTCATTTGGAGAGAACACGGGGGACTGAATTCGTCGACTTTGCGGCCGCATGGCTGAGCATTATGGTCAACAAC

Pro_35Sx2 SiOleosinL (opt)

760 780 800 820 840

AGCAGACCAGGGCGCCTCACCTGCAGCTGCAGCCGCGCGCCCAGCGGGTAGTGAAGGCGGCCACCGCCGTGACAGCCGGCGGCTCGCTTCTCGTCCTCTCTGGCCTC

SiOleosinL (opt)

860 880 900 920 940 960

ACTTTAGCCGGAACTGTTATTGCGCTCACCATCGCCACTCCGCTGCTTGTGATCTTTAGCCCCGTTCTGGTGCCGGCGGTCATAACCATTTTCTTGCTGGGTGCGGG

SiOleosinL (opt)

980 1,000 1,020 1,040 1,060

pOIL382: Nb-optimized SiOleosinL in pJP3343 (7507 bp) (from 1071-21...

TTTTCTGGCATCCGGAGGCTTCGGCGTGGCGGCGCTGAGTGTGCTGTCGTGGATTTACAGATATCTGACAGGGAAACACCCGCCGGGGGCGGATCAGCTGGAATCGG

SiOleosinL (opt)

1,080 1,100 1,120 1,140 1,160

CAAAGACGAAGCTGGCGAGCAAGGCGCGAGAGATGAAGGATAGGGCAGAGCAGTTCTCGCAGCAGCCTGTTGCGGGGTCTCAAACTTCTTGAGAGCTCGGTACCTTT

SiOleosinL (opt)

1,180 1,200 1,220 1,240 1,260 1,280

TACTAGTGATATCCCTGTGTGAAATTGTTATCCGCTAGCGTGATCGTTCAAACATTTGGCAATAAAGTTTCTTAAGATTGAATCCTGTTGCCGGTCTTGCGATGATT

NOS terminator

1,300 1,320 1,340 1,360 1,380

ATCATATAATTTCTGTTGAATTACGTTAAGCATGTAATAATTAACATGTAATGCATGACGTTATTTATGAGATGGGTTTTTATGATTAGAGTCCCGCAATTATACAT

NOS terminator

1,400 1,420 1,440 1,460 1,480

TTAATACGCGATAGAAAACAAAATATAGCGCGCAAACTAGGATAAATTATCGCGCGCGGTGTCATCTATGTTACTAGATCCCATGGGAAGTTCCTATTCCGAAGTTC

NOS terminator

FRT site

1,500 1,520 1,540 1,560 1,580 1,600

CTATTCTCTGAAAAGTATAGGAACTTCAGCGATCGCAGACGTCGGGATCTTCTGCAAGCATCTCTATTTCCTGAAGGTCTAACCTCGAAGATTTAAGATTTAATTAC

FRT site

enTCUP2 promoter

1,620 1,640 1,660 1,680 1,700

GTTTATAATTACAAAATTGATTCTAGTATCTTTAATTTAATGCTTATACATTATTAATTAATTTAGTACTTTCAATTTGTTTTCAGAAATTATTTTACTATTTTTTA

enTCUP2 promoter

1,720 1,740 1,760 1,780 1,800

TAAAATAAAAGGGAGAAAATGGCTATTTAAATACTAGCCTATTTTATTTCAATTTTAGCTTAAAATCAGCCCCAATTAGCCCCAATTTCAAATTCAAATGGTCCAGC

enTCUP2 promoter

1,820 1,840 1,860 1,880 1,900 1,920

CCAATTCCTAAATAACCCACCCCTAACCCGCCCGGTTTCCCCTTTTGATCCATGCAGTCAACGCCCAGAATTTCCCTATATAATTTTTTAATTCCCAAACACCCCTA

enTCUP2 promoter

1,940 1,960 1,980 2,000 2,020

ACTCTATCCCATTTCTCACCAACCGCCACATAGATCTATCCTCTTATCTCTCAAACTCTCTCGAACCTTCCCCTAACCCTAGCAGCCTCTCATCATCCTCACCTCAA

enTCUP2 promoter

2,040 2,060 2,080 2,100 2,120 2,140

pOIL382: Nb-optimized SiOleosinL in pJP3343 (7507 bp) (from 2141-32...

AACCCACCGGGGCCGGCCATGATTGAACAAGATGGATTGCACGCAGGTTCTCCGGCCGCTTGGGTGGAGAGGCTATTCGGCTATGACTGGGCACAACAGACAATCGG

en...r neomycin phosphotransferase II

2,160 2,180 2,200 2,220 2,240

CTGCTCTGATGCCGCCGTGTTCCGGCTGTCAGCGCAGGGGAGGCCGGTTCTTTTTGTCAAGACCGACCTGTCCGGTGCCCTGAATGAACTTCAAGACGAGGCAGCGC

neomycin phosphotransferase II

2,260 2,280 2,300 2,320 2,340

GGCTATCGTGGCTGGCCACGACGGGCGTTCCTTGCGCAGCTGTGCTCGACGTTGTCACTGAAGCGGGAAGGGACTGGCTGCTATTGGGCGAAGTGCCGGGGCAGGAT

neomycin phosphotransferase II

2,360 2,380 2,400 2,420 2,440 2,460

CTCCTGTCATCTCACCTTGCTCCTGCCGAGAAAGTATCCATCATGGCTGATGCAATGCGGCGGCTGCATACGCTTGATCCGGCTACCTGCCCATTCGACCACCAAGC

neomycin phosphotransferase II

2,480 2,500 2,520 2,540 2,560

GAAACATCGCATCGAGCGAGCACGTACTCGGATGGAAGCCGGTCTTGTCGATCAGGATGATCTGGACGAAGAGCATCAGGGGCTCGCGCCAGCCGAACTGTTCGCCA

neomycin phosphotransferase II

2,580 2,600 2,620 2,640 2,660

GGCTCAAGGCGCGCATGCCCGACGGCGAGGATCTCGTCGTGACTCATGGCGATGCCTGCTTGCCGAATATCATGGTGGAAAATGGCCGCTTTTCTGGATTCATCGAC

neomycin phosphotransferase II

2,680 2,700 2,720 2,740 2,760 2,780

TGTGGCCGGCTGGGTGTGGCGGACCGCTATCAGGACATAGCGTTGGCTACCCGTGATATTGCTGAAGAGCTTGGCGGCGAATGGGCTGACCGCTTCCTCGTGCTTTA

neomycin phosphotransferase II

2,800 2,820 2,840 2,860 2,880

CGGTATCGCCGCTCCCGATTCGCAGCGCATCGCCTTCTATCGCCTTCTTGACGAGTTCTTCTGAGGCGCGCCGATCGTTCAAACATTTGGCAATAAAGTTTCTTAAG

neomycin phosphotransferase II NOS terminator

2,900 2,920 2,940 2,960 2,980

ATTGAATCCTGTTGCCGGTCTTGCGATGATTATCATATAATTTCTGTTGAATTACGTTAAGCATGTAATAATTAACATGTAATGCATGACGTTATTTATGAGATGGG

NOS terminator

3,000 3,020 3,040 3,060 3,080 3,100

TTTTTATGATTAGAGTCCCGCAATTATACATTTAATACGCGATAGAAAACAAAATATAGCGCGCAAACTAGGATAAATTATCGCGCGCGGTGTCATCTATGTTACTA

NOS terminator

3,120 3,140 3,160 3,180 3,200

pOIL382: Nb-optimized SiOleosinL in pJP3343 (7507 bp) (from 3211-43...

GATCCCTAGGGAAGTTCCTATTCCGAAGTTCCTATTCTCTGAAAAGTATAGGAACTTCTTTGCGTATTGGGCGCTCTTGGCCTTTTTGGCCACCGGTCGTACGGTTA

FRT site

3,220 3,240 3,260 3,280 3,300

AAACCACCCCAGTACATTAAAAACGTCCGCAATGTGTTATTAAGTTGTCTAAGCGTCAATTTGTTTACACCACAATATATCCTGCCACCAGCCAGCCAACAGCTCCC

T-DNA left border

3,320 3,340 3,360 3,380 3,400 3,420

CGACCGGCAGCTCGGCACAAAATCACCACTCGATACAGGCAGCCCATCAGTCCACTAGACGCTCACCGGGCTGGTTGCCCTCGCCGCTGGGCTGGCGGCCGTCTATG

T-DNA left border RK2 oriV

3,440 3,460 3,480 3,500 3,520

GCCCTGCAAACGCGCCAGAAACGCCGTCGAAGCCGTGTGCGAGACACCGCAGCCGCCGGCGTTGTGGATACCTCGCGGAAAACTTGGCCCTCACTGACAGATGAGGG

RK2 oriV

3,540 3,560 3,580 3,600 3,620

GCGGACGTTGACACTTGAGGGGCCGACTCACCCGGCGCGGCGTTGACAGATGAGGGGCAGGCTCGATTTCGGCCGGCGACGTGGAGCTGGCCAGCCTCGCAAATCGG

RK2 oriV

3,640 3,660 3,680 3,700 3,720 3,740

CGAAAACGCCTGATTTTACGCGAGTTTCCCACAGATGATGTGGACAAGCCTGGGGATAAGTGCCCTGCGGTATTGACACTTGAGGGGCGCGACTACTGACAGATGAG

RK2 oriV

3,760 3,780 3,800 3,820 3,840

GGGCGCGATCCTTGACACTTGAGGGGCAGAGTGCTGACAGATGAGGGGCGCACCTATTGACATTTGAGGGGCTGTCCACAGGCAGAAAATCCAGCATTTGCAAGGGT

RK2 oriV

3,860 3,880 3,900 3,920 3,940

TTCCGCCCGTTTTTCGGCCACCGCTAACCTGTCTTTTAACCTGCTTTTAAACCAATATTTATAAACCTTGTTTTTAACCAGGGCTGCGCCCTGTGCGCGTGACCGCG

RK2 oriV

3,960 3,980 4,000 4,020 4,040 4,060

CACGCCGAAGGGGGGTGCCCCCCCTTCTCGAACCCTCCCGGCCCGCTCTCGCGTTGGCAGCATCACCCATAATTGTGGTTTCAAAATCGGCTCCGTCGATACTATGT

RK2 oriV

4,080 4,100 4,120 4,140 4,160

TATACGCCAACTTTGAAAACAACTTTGAAAAAGCTGTTTTCTGGTATTTAAGGTTTTAGAATGCAAGGAACAGTGAATTGGAGTTCGTCTTGTTATAATTAGCTTCT

4,180 4,200 4,220 4,240 4,260 4,280

TGGGGTATTTAAATACTGTAGAAAAGAGGAAGGAAATAATAAATGGCTAAAATGAGAATATCACCGGAATTGAAAAAACTGATCGAAAAATACCGCTGCGTAAAAGA

neomycin-kanamycin phosphotransferase type III

4,300 4,320 4,340 4,360 4,380

pOIL382: Nb-optimized SiOleosinL in pJP3343 (7507 bp) (from 4388-55...

TACGGAAGGAATGTCTCCTGCTAAGGTATATAAGCTGGTGGGAGAAAATGAAAACCTATATTTAAAAATGACGGACAGCCGGTATAAAGGGACCACCTATGATGTGG

neomycin-kanamycin phosphotransferase type III

4,400 4,420 4,440 4,460 4,480

AACGGGAAAAGGACATGATGCTATGGCTGGAAGGAAAGCTGCCTGTTCCAAAGGTCCTGCACCTTGAACGGCATGATGGCTGGAGCAATCTGCTCATGAGTGAGGCC

neomycin-kanamycin phosphotransferase type III

4,500 4,520 4,540 4,560 4,580 4,600

GATGGCGTCCTTTGCTCGGAAGAGTATGAAGATGAACAAAGCCCTGAAAAGATTATCGAGCTGTATGCGGAGTGCATCAGGCTCTTTCACTCCATCGACATATCGGA

neomycin-kanamycin phosphotransferase type III

4,620 4,640 4,660 4,680 4,700

TTGTCCCTATACGAATAGCTTAGACAGCCGCTTAGCCGAATTGGATTACTTACTGAATAACGATCTGGCCGATGTGGATTGCGAAAACTGGGAAGAAGACACCCCAT

neomycin-kanamycin phosphotransferase type III

4,720 4,740 4,760 4,780 4,800

TTAAAGATCCGCGCGAGCTGTATGATTTTTTAAAGACGGAAAAGCCCGAAGAGGAACTTGTCTTTTCCCACGGCGACCTGGGAGACAGCAACATCTTTGTGAAAGAT

neomycin-kanamycin phosphotransferase type III

4,820 4,840 4,860 4,880 4,900 4,920

GGCAAAGTAAGTGGCTTTATTGATCTTGGGAGAAGCGGCAGGGCGGACAAGTGGTATGACATTGCCTTCTGCGTCCGGTCGATCAGGGAGGATATTGGGGAAGAACA

neomycin-kanamycin phosphotransferase type III

4,940 4,960 4,980 5,000 5,020

GTATGTCGAGCTATTTTTTGACTTACTGGGGATCAAGCCTGATTGGGAGAAAATAAAATATTATATTTTACTGGATGAATTGTTTTAGTACCTAGATGTGGCGCAAC

neomycin-kanamycin phosphotransferase type III

5,040 5,060 5,080 5,100 5,120

GATGCTGGCGACAAGCAGGAGCGCACCGACTTCTTCCGCATCAAGTGTTTTGGCTCTCAGGCCGAGGCCCACGGCAAGTATTTGGGCAAGGGGTCGCTGGTATTCGT

5,140 5,160 5,180 5,200 5,220 5,240

GCAGGGCAAGATTCGGAATACCAAGTACGAGAAGGACGGCCAGACGGTCTACGGGACCGACTTCATTGCCGATAAGGTGGATTATCTGGACACCAAGGCACCAGGCG

5,260 5,280 5,300 5,320 5,340

GATCAAATCAGGAATAAGGGCACATTGCCCCGGCGTGAGTCGGGGCAATCCCGCAAGGAGGGTGAATGAATCGGACGTTTGACCGGAAGGCATACAGGCAAGAACTG

derived from plasmid RK2

5,360 5,380 5,400 5,420 5,440

ATCGACGCGGGGTTTTCCGCCGAGGATGCCGAAACCATCGCAAGCCGCACCGTCATGCGTGCGCCCCGCGAAACCTTCCAGTCCGTCGGCTCGATGGCCCAGCAAGC

derived from plasmid RK2

5,460 5,480 5,500 5,520 5,540 5,560

TACGGCCAAGATCGAGCGCGACAGCGTGCAACTGGCTCCCCCTGCCCTGCCCGCGCCATCGGCCGCCGTGGAGCGTTCGCGTCGTCTCGAACAGGAGGCGGCAGGTT

derived from plasmid RK2

5,580 5,600 5,620 5,640 5,660

TGGCGAAGTCGATGACCATCGACACGCGAGGAACTATGACGACCAAGAAGCGAAAAACCGCCGGCGAGGACCTGGCAAAACAGGTCAGCGAGGCCAAGCAAGCCGCG

derived from plasmid RK2

5,680 5,700 5,720 5,740 5,760

TTGCTGAAACACACGAAGCAGCAGATCAAGGAAATGCAGCTTTCCTTGTTCGATATTGCGCCGTGGCCGGACACGATGCGAGCGATGCCAAACGACACGGCCCGCTC

derived from plasmid RK2

5,780 5,800 5,820 5,840 5,860 5,880

TGCCCTGTTCACCACGCGCAACAAGAAAATCCCGCGCGAGGCGCTGCAAAACAAGGTCATTTTCCACGTCAACAAGGACGTGAAGATCACCTACACCGGCGTCGAGC

derived from plasmid RK2

5,900 5,920 5,940 5,960 5,980

TGCGGGCCGACGATGACGAACTGGTGTGGCAGCAGGTGTTGGAGTACGCGAAGCGCACCCCTATCGGCGAGCCGATCACCTTCACGTTCTACGAGCTTTGCCAGGAC

derived from plasmid RK2

6,000 6,020 6,040 6,060 6,080

CTGGGCTGGTCGATCAATGGCCGGTATTACACGAAGGCCGAGGAATGCCTGTCGCGCCTACAGGCGACGGCGATGGGCTTCACGTCCGACCGCGTTGGGCACCTGGA

derived from plasmid RK2

6,100 6,120 6,140 6,160 6,180 6,200

ATCGGTGTCGCTGCTGCACCGCTTCCGCGTCCTGGACCGTGGCAAGAAAACGTCCCGTTGCCAGGTCCTGATCGACGAGGAAATCGTCGTGCTGTTTGCTGGCGACC

derived from plasmid RK2

6,220 6,240 6,260 6,280 6,300

ACTACACGAAATTCATATGGGAGAAGTACCGCAAGCTGTCGCCGACGGCCCGACGGATGTTCGACTATTTCAGCTCGCACCGGGAGCCGTACCCGCTCAAGCTGGAA

derived from plasmid RK2

6,320 6,340 6,360 6,380 6,400 6,420

ACCTTCCGCCTCATGTGCGGATCGGATTCCACCCGCGTGAAGAAGTGGCGCGAGCAGGTCGGCGAAGCCTGCGAAGAGTTGCGAGGCAGCGGCCTGGTGGAACACGC

derived from plasmid RK2

6,440 6,460 6,480 6,500 6,520

CTGGGTCAATGATGACCTGGTGCATTGCAAACGCTAGGGCCTTGTGGGGTCAGTTCCGGCTGGGGGTTCAGCAGCCAGCGCTTTACTGAGATCCTCTTCCGCTTCCT

derived from plasmid RK2 ColE1

6,540 6,560 6,580 6,600 6,620

pOIL382: Nb-optimized SiOleosinL in pJP3343 (7507 bp) (from 6635-75...

CGCTCACTGACTCGCTGCGCTCGGTCGTTCGGCTGCGGCGAGCGGTATCAGCTCACTCAAAGGCGGTAATACGGTTATCCACAGAATCAGGGGATAACGCAGGAAAG

ColE1

6,640 6,660 6,680 6,700 6,720 6,740

AACATGTGAGCAAAAGGCCAGCAAAAGGCCAGGAACCGTAAAAAGGCCGCGTTGCTGGCGTTTTTCCATAGGCTCCGCCCCCCTGACGAGCATCACAAAAATCGACG

ColE1

6,760 6,780 6,800 6,820 6,840

CTCAAGTCAGAGGTGGCGAAACCCGACAGGACTATAAAGATACCAGGCGTTTCCCCCTGGAAGCTCCCTCGTGCGCTCTCCTGTTCCGACCCTGCCGCTTACCGGAT

ColE1

6,860 6,880 6,900 6,920 6,940

ACCTGTCCGCCTTTCTCCCTTCGGGAAGCGTGGCGCTTTCTCATAGCTCACGCTGTAGGTATCTCAGTTCGGTGTAGGTCGTTCGCTCCAAGCTGGGCTGTGTGCAC

ColE1

6,960 6,980 7,000 7,020 7,040 7,060

GAACCCCCCGTTCAGCCCGACCGCTGCGCCTTATCCGGTAACTATCGTCTTGAGTCCAACCCGGTAAGACACGACTTATCGCCACTGGCAGCAGCCACTGGTAACAG

ColE1

7,080 7,100 7,120 7,140 7,160

GATTAGCAGAGCGAGGTATGTAGGCGGTGCTACAGAGTTCTTGAAGTGGTGGCCTAACTACGGCTACACTAGAAGAACAGTATTTGGTATCTGCGCTCTGCTGAAGC

ColE1

7,180 7,200 7,220 7,240 7,260

CAGTTACCTTCGGAAAAAGAGTTGGTAGCTCTTGATCCGGCAAACAAACCACCGCTGGTAGCGGTGGTTTTTTTGTTTGCAAGCAGCAGATTACGCGCAGAAAAAAA

ColE1

7,280 7,300 7,320 7,340 7,360 7,380

GGATCTCAAGAAGATCCTTTGATCTTTTCTACGGGGTCTGACGCTCAGTGGAACGAAAACTCACGTTAAGGGATTTTGGTCATGAGATTATCAAAAAGGATCTTCAC

ColE1

7,400 7,420 7,440 7,460 7,480

CTAGATCCTTTTGGATC

ColE1

7,500

(from 1-1070 bp)

pOIL383: Nb-optimized SiOleosinH in pJP3343

TCCTGTGGTTGGCATGCACATACAAATGGACGAACGGATAAACCTTTTCACGCCCTTTTAAATATCCGATTATTCTAATAAACGCTCTTTTCTCTTAGGTTTACCCG

T-DNA right border

20 40 60 80 100

CCAATATATCCTGTCAAACACTGATAGTTTAAACTGAAGGCGGGAAACGACAATCTGCTAGTGGATCTCCCAGTCACGACGTTGTAAAACGGGCGCCCCGCGGAAAG

T-DNA right border

120 140 160 180 200

CTTGCTAGCCAATTGGGGCCCAACGTTCTCGAGTTTTTCTAGAAGGCCTTGGATCCCATGGAGTCAAAGATTCAAATAGAGGACCTAACAGAACTCGCCGTAAAGAC

Pro_35Sx2

220 240 260 280 300 320

TGGCGAACAGTTCATACAGAGTCTCTTACGACTCAATGACAAGAAGAAAATCTTCGTCAACATGGTGGAGCACGACACACTTGTCTACTCCAAAAATATCAAAGATA

Pro_35Sx2

340 360 380 400 420

CAGTCTCAGAAGACCAAAGGGCAATTGAGACTTTTCAACAAAGGGTAATATCCGGAAACCTCCTCGGATTCCATTGCCCAGCTATCTGTCACTTTATTGTGAAGATA

Pro_35Sx2

440 460 480 500 520

GTGGAAAAGGAAGGTGGCTCCTACAAATGCCATCATTGCGATAAAGGAAAGGCCATCGTTGAAGATGCCTCTGCCGACAGTGGTCCCAAAGATGGACCCCCACCCAC

Pro_35Sx2

540 560 580 600 620 640

GAGGAGCATCGTGGAAAAAGAAGACGTTCCAACCACGTCTTCAAAGCAAGTGGATTGATGTGATATCTCCACTGACGTAAGGGATGACGCACAATCCCACTATCCTT

Pro_35Sx2

660 680 700 720 740

CGCAAGACCCTTCCTCTATATAAGGAAGTTCATTTCATTTGGAGAGAACACGGGGGACTGAATTCGTCGACTTTGCGGCCGCATGGCGGACCGCGACCGTCCACACC

Pro_35Sx2 SiOleosinH (opt)

760 780 800 820 840

CCCACCAAATTCAAGTCCACCCTCAACATCCGCACCGCTATGAAGGTGGCGTCAAGTCTCTCCTCCCTCAAAAGGGCCCCTCCACCACCCAGATTCTCGCCATAATC

SiOleosinH (opt)

860 880 900 920 940 960

ACCCTCCTTCCCATCAGCGGCACGCTTCTTTGCCTAGCTGGGATCACGCTCGTCGGGACCCTCATCGGACTTGCAGTCGCCACCCCAGTCTTCGTGATCTTCAGCCC

SiOleosinH (opt)

980 1,000 1,020 1,040 1,060

pOIL383: Nb-optimized SiOleosinH in pJP3343 (7570 bp) (from 1071-21...

TGTTCTGGTTCCCGCAGCCATACTGATAGCCGGCGCGGTCACGGCGTTTTTGACGTCCGGGGCTTTTGGGCTGACGGGGCTTTCGTCGCTTTCTTGGGTTCTGAATT

SiOleosinH (opt)

1,080 1,100 1,120 1,140 1,160

CATTCAGACGGGCGACGGGGCAGGGGCCGTTGGAGTACGCGAAGCGAGGCGTGCAGGAGGGGACTTTGTATGTGGGAGAGAAGACGAAGCAAGCGGGCGAAGCGATT

SiOleosinH (opt)

1,180 1,200 1,220 1,240 1,260 1,280

AAGAGCACAGCCAAGGAAGGAGGGCGAGAAGGGACTGCACGGACTTGAGAGCTCGGTACCTTTTACTAGTGATATCCCTGTGTGAAATTGTTATCCGCTAGCGTGAT

SiOleosinH (opt)

1,300 1,320 1,340 1,360 1,380

CGTTCAAACATTTGGCAATAAAGTTTCTTAAGATTGAATCCTGTTGCCGGTCTTGCGATGATTATCATATAATTTCTGTTGAATTACGTTAAGCATGTAATAATTAA

NOS terminator

1,400 1,420 1,440 1,460 1,480

CATGTAATGCATGACGTTATTTATGAGATGGGTTTTTATGATTAGAGTCCCGCAATTATACATTTAATACGCGATAGAAAACAAAATATAGCGCGCAAACTAGGATA

NOS terminator

1,500 1,520 1,540 1,560 1,580 1,600

AATTATCGCGCGCGGTGTCATCTATGTTACTAGATCCCATGGGAAGTTCCTATTCCGAAGTTCCTATTCTCTGAAAAGTATAGGAACTTCAGCGATCGCAGACGTCG

NOS terminator FRT site

1,620 1,640 1,660 1,680 1,700

GGATCTTCTGCAAGCATCTCTATTTCCTGAAGGTCTAACCTCGAAGATTTAAGATTTAATTACGTTTATAATTACAAAATTGATTCTAGTATCTTTAATTTAATGCT

enTCUP2 promoter

1,720 1,740 1,760 1,780 1,800

TATACATTATTAATTAATTTAGTACTTTCAATTTGTTTTCAGAAATTATTTTACTATTTTTTATAAAATAAAAGGGAGAAAATGGCTATTTAAATACTAGCCTATTT

enTCUP2 promoter

1,820 1,840 1,860 1,880 1,900 1,920

TATTTCAATTTTAGCTTAAAATCAGCCCCAATTAGCCCCAATTTCAAATTCAAATGGTCCAGCCCAATTCCTAAATAACCCACCCCTAACCCGCCCGGTTTCCCCTT

enTCUP2 promoter

1,940 1,960 1,980 2,000 2,020

TTGATCCATGCAGTCAACGCCCAGAATTTCCCTATATAATTTTTTAATTCCCAAACACCCCTAACTCTATCCCATTTCTCACCAACCGCCACATAGATCTATCCTCT

enTCUP2 promoter

2,040 2,060 2,080 2,100 2,120 2,140

pOIL383: Nb-optimized SiOleosinH in pJP3343 (7570 bp) (from 2141-32...

TATCTCTCAAACTCTCTCGAACCTTCCCCTAACCCTAGCAGCCTCTCATCATCCTCACCTCAAAACCCACCGGGGCCGGCCATGATTGAACAAGATGGATTGCACGC

enTCUP2 promoter | neomycin p...ferase II

2,160 2,180 2,200 2,220 2,240

AGGTTCTCCGGCCGCTTGGGTGGAGAGGCTATTCGGCTATGACTGGGCACAACAGACAATCGGCTGCTCTGATGCCGCCGTGTTCCGGCTGTCAGCGCAGGGGAGGC

neomycin phosphotransferase II

2,260 2,280 2,300 2,320 2,340

CGGTTCTTTTTGTCAAGACCGACCTGTCCGGTGCCCTGAATGAACTTCAAGACGAGGCAGCGCGGCTATCGTGGCTGGCCACGACGGGCGTTCCTTGCGCAGCTGTG

neomycin phosphotransferase II

2,360 2,380 2,400 2,420 2,440 2,460

CTCGACGTTGTCACTGAAGCGGGAAGGGACTGGCTGCTATTGGGCGAAGTGCCGGGGCAGGATCTCCTGTCATCTCACCTTGCTCCTGCCGAGAAAGTATCCATCAT

neomycin phosphotransferase II

2,480 2,500 2,520 2,540 2,560

GGCTGATGCAATGCGGCGGCTGCATACGCTTGATCCGGCTACCTGCCCATTCGACCACCAAGCGAAACATCGCATCGAGCGAGCACGTACTCGGATGGAAGCCGGTC

neomycin phosphotransferase II

2,580 2,600 2,620 2,640 2,660

TTGTCGATCAGGATGATCTGGACGAAGAGCATCAGGGGCTCGCGCCAGCCGAACTGTTCGCCAGGCTCAAGGCGCGCATGCCCGACGGCGAGGATCTCGTCGTGACT

neomycin phosphotransferase II

2,680 2,700 2,720 2,740 2,760 2,780

CATGGCGATGCCTGCTTGCCGAATATCATGGTGGAAAATGGCCGCTTTTCTGGATTCATCGACTGTGGCCGGCTGGGTGTGGCGGACCGCTATCAGGACATAGCGTT

neomycin phosphotransferase II

2,800 2,820 2,840 2,860 2,880

GGCTACCCGTGATATTGCTGAAGAGCTTGGCGGCGAATGGGCTGACCGCTTCCTCGTGCTTTACGGTATCGCCGCTCCCGATTCGCAGCGCATCGCCTTCTATCGCC

neomycin phosphotransferase II

2,900 2,920 2,940 2,960 2,980

TTCTTGACGAGTTCTTCTGAGGCGCGCCGATCGTTCAAACATTTGGCAATAAAGTTTCTTAAGATTGAATCCTGTTGCCGGTCTTGCGATGATTATCATATAATTTC

neomyci...ase II | NOS terminator

3,000 3,020 3,040 3,060 3,080 3,100

TGTTGAATTACGTTAAGCATGTAATAATTAACATGTAATGCATGACGTTATTTATGAGATGGGTTTTTATGATTAGAGTCCCGCAATTATACATTTAATACGCGATA

NOS terminator

3,120 3,140 3,160 3,180 3,200

pOIL383: Nb-optimized SiOleosinH in pJP3343 (7570 bp) (from 3211-42...

GAAAACAAAATATAGCGCGCAAACTAGGATAAATTATCGCGCGCGGTGTCATCTATGTTACTAGATCCCTAGGGAAGTTCCTATTCCGAAGTTCCTATTCTCTGAAA

NOS terminator   FRT site

3,220 3,240 3,260 3,280 3,300

AGTATAGGAACTTCTTTGCGTATTGGGCGCTCTTGGCCTTTTTGGCCACCGGTCGTACGGTTAAAACCACCCCAGTACATTAAAAACGTCCGCAATGTGTTATTAAG

FRT site   T-DNA left border

3,320 3,340 3,360 3,380 3,400 3,420

TTGTCTAAGCGTCAATTTGTTTACACCACAATATATCCTGCCACCAGCCAGCCAACAGCTCCCCGACCGGCAGCTCGGCACAAAATCACCACTCGATACAGGCAGCC

T-DNA left border

3,440 3,460 3,480 3,500 3,520

CATCAGTCCACTAGACGCTCACCGGGCTGGTTGCCCTCGCCGCTGGGCTGGCGGCCGTCTATGGCCCTGCAAACGCGCCAGAAACGCCGTCGAAGCCGTGTGCGAGA

RK2 oriV

3,540 3,560 3,580 3,600 3,620

CACCGCAGCCGCCGGCGTTGTGGATACCTCGCGGAAAACTTGGCCCTCACTGACAGATGAGGGGCGGACGTTGACACTTGAGGGGCCGACTCACCCGGCGCGGCGTT

RK2 oriV

3,640 3,660 3,680 3,700 3,720 3,740

GACAGATGAGGGGCAGGCTCGATTTCGGCCGGCGACGTGGAGCTGGCCAGCCTCGCAAATCGGCGAAAACGCCTGATTTTACGCGAGTTTCCCACAGATGATGTGGA

RK2 oriV

3,760 3,780 3,800 3,820 3,840

CAAGCCTGGGGATAAGTGCCCTGCGGTATTGACACTTGAGGGGCGCGACTACTGACAGATGAGGGGCGCGATCCTTGACACTTGAGGGGCAGAGTGCTGACAGATGA

RK2 oriV

3,860 3,880 3,900 3,920 3,940

GGGGCGCACCTATTGACATTTGAGGGGCTGTCCACAGGCAGAAAATCCAGCATTTGCAAGGGTTTCCGCCCGTTTTTCGGCCACCGCTAACCTGTCTTTTAACCTGC

RK2 oriV

3,960 3,980 4,000 4,020 4,040 4,060

TTTTAAACCAATATTTATAAACCTTGTTTTTAACCAGGGCTGCGCCCTGTGCGCGTGACCGCGCACGCCGAAGGGGGGTGCCCCCCCTTCTCGAACCCTCCCGGCCC

RK2 oriV

4,080 4,100 4,120 4,140 4,160

GCTCTCGCGTTGGCAGCATCACCCATAATTGTGGTTTCAAAATCGGCTCCGTCGATACTATGTTATACGCCAACTTTGAAAACAACTTTGAAAAAGCTGTTTTCTGG

4,180 4,200 4,220 4,240 4,260 4,280

pOIL383: Nb-optimized SiOleosinH in pJP3343 (7570 bp) (from 4281-54...

TATTTAAGGTTTTAGAATGCAAGGAACAGTGAATTGGAGTTCGTCTTGTTATAATTAGCTTCTTGGGGTATTTAAATACTGTAGAAAAGAGGAAGGAAATAATAAAT

4,300 4,320 4,340 4,360 4,380

GGCTAAAATGAGAATATCACCGGAATTGAAAAAACTGATCGAAAAATACCGCTGCGTAAAAGATACGGAAGGAATGTCTCCTGCTAAGGTATATAAGCTGGTGGGAG

neomycin-kanamycin phosphotransferase type III

4,400 4,420 4,440 4,460 4,480

AAAATGAAAACCTATATTTAAAAATGACGGACAGCCGGTATAAAGGGACCACCTATGATGTGGAACGGGAAAAGGACATGATGCTATGGCTGGAAGGAAAGCTGCCT

neomycin-kanamycin phosphotransferase type III

4,500 4,520 4,540 4,560 4,580 4,600

GTTCCAAAGGTCCTGCACCTTGAACGGCATGATGGCTGGAGCAATCTGCTCATGAGTGAGGCCGATGGCGTCCTTTGCTCGGAAGAGTATGAAGATGAACAAAGCCC

neomycin-kanamycin phosphotransferase type III

4,620 4,640 4,660 4,680 4,700

TGAAAAGATTATCGAGCTGTATGCGGAGTGCATCAGGCTCTTTCACTCCATCGACATATCGGATTGTCCCTATACGAATAGCTTAGACAGCCGCTTAGCCGAATTGG

neomycin-kanamycin phosphotransferase type III

4,720 4,740 4,760 4,780 4,800

ATTACTTACTGAATAACGATCTGGCCGATGTGGATTGCGAAAACTGGGAAGAAGACACCCCATTTAAAGATCCGCGCGAGCTGTATGATTTTTTAAAGACGGAAAAG

neomycin-kanamycin phosphotransferase type III

4,820 4,840 4,860 4,880 4,900 4,920

CCCGAAGAGGAACTTGTCTTTTCCCACGGCGACCTGGGAGACAGCAACATCTTTGTGAAAGATGGCAAAGTAAGTGGCTTTATTGATCTTGGGAGAAGCGGCAGGGC

neomycin-kanamycin phosphotransferase type III

4,940 4,960 4,980 5,000 5,020

GGACAAGTGGTATGACATTGCCTTCTGCGTCCGGTCGATCAGGGAGGATATTGGGGAAGAACAGTATGTCGAGCTATTTTTTGACTTACTGGGGATCAAGCCTGATT

neomycin-kanamycin phosphotransferase type III

5,040 5,060 5,080 5,100 5,120

GGGAGAAAATAAAATATTATATTTTACTGGATGAATTGTTTTAGTACCTAGATGTGGCGCAACGATGCTGGCGACAAGCAGGAGCGCACCGACTTCTTCCGCATCAA

neomycin-kanamycin ...ansferase type III

5,140 5,160 5,180 5,200 5,220 5,240

GTGTTTTGGCTCTCAGGCCGAGGCCCACGGCAAGTATTTGGGCAAGGGGTCGCTGGTATTCGTGCAGGGCAAGATTCGGAATACCAAGTACGAGAAGGACGGCCAGA

5,260 5,280 5,300 5,320 5,340

CGGTCTACGGGACCGACTTCATTGCCGATAAGGTGGATTATCTGGACACCAAGGCACCAGGCGGATCAAATCAGGAATAAGGGCACATTGCCCCGGCGTGAGTCGGG

5,360 5,380 5,400 5,420 5,440

GCAATCCCGCAAGGAGGGTGAATGAATCGGACGTTTGACCGGAAGGCATACAGGCAAGAACTGATCGACGCGGGGTTTTCCGCCGAGGATGCCGAAACCATCGCAAG

derived from plasmid RK2

5,460 5,480 5,500 5,520 5,540 5,560

CCGCACCGTCATGCGTGCGCCCCGCGAAACCTTCCAGTCCGTCGGCTCGATGGCCCAGCAAGCTACGGCCAAGATCGAGCGCGACAGCGTGCAACTGGCTCCCCCTG

derived from plasmid RK2

5,580 5,600 5,620 5,640 5,660

CCCTGCCCGCGCCATCGGCCGCCGTGGAGCGTTCGCGTCGTCTCGAACAGGAGGCGGCAGGTTTGGCGAAGTCGATGACCATCGACACGCGAGGAACTATGACGACC

derived from plasmid RK2

5,680 5,700 5,720 5,740 5,760

AAGAAGCGAAAAACCGCCGGCGAGGACCTGGCAAAACAGGTCAGCGAGGCCAAGCAAGCCGCGTTGCTGAAACACACGAAGCAGCAGATCAAGGAAATGCAGCTTTC

derived from plasmid RK2

5,780 5,800 5,820 5,840 5,860 5,880

CTTGTTCGATATTGCGCCGTGGCCGGACACGATGCGAGCGATGCCAAACGACACGGCCCGCTCTGCCCTGTTCACCACGCGCAACAAGAAAATCCCGCGCGAGGCGC

derived from plasmid RK2

5,900 5,920 5,940 5,960 5,980

TGCAAAACAAGGTCATTTTCCACGTCAACAAGGACGTGAAGATCACCTACACCGGCGTCGAGCTGCGGGCCGACGATGACGAACTGGTGTGGCAGCAGGTGTTGGAG

derived from plasmid RK2

6,000 6,020 6,040 6,060 6,080

TACGCGAAGCGCACCCCTATCGGCGAGCCGATCACCTTCACGTTCTACGAGCTTTGCCAGGACCTGGGCTGGTCGATCAATGGCCGGTATTACACGAAGGCCGAGGA

derived from plasmid RK2

6,100 6,120 6,140 6,160 6,180 6,200

ATGCCTGTCGCGCCTACAGGCGACGGCGATGGGCTTCACGTCCGACCGCGTTGGGCACCTGGAATCGGTGTCGCTGCTGCACCGCTTCCGCGTCCTGGACCGTGGCA

derived from plasmid RK2

6,220 6,240 6,260 6,280 6,300

AGAAAACGTCCCGTTGCCAGGTCCTGATCGACGAGGAAATCGTCGTGCTGTTTGCTGGCGACCACTACACGAAATTCATATGGGAGAAGTACCGCAAGCTGTCGCCG

derived from plasmid RK2

6,320 6,340 6,360 6,380 6,400 6,420

ACGGCCCGACGGATGTTCGACTATTTCAGCTCGCACCGGGAGCCGTACCCGCTCAAGCTGGAAACCTTCCGCCTCATGTGCGGATCGGATTCCACCCGCGTGAAGAA

derived from plasmid RK2

6,440 6,460 6,480 6,500 6,520

pOIL383: Nb-optimized SiOleosinH in pJP3343 (7570 bp) (from 6528-75...

GTGGCGCGAGCAGGTCGGCGAAGCCTGCGAAGAGTTGCGAGGCAGCGGCCTGGTGGAACACGCCTGGGTCAATGATGACCTGGTGCATTGCAAACGCTAGGGCCTTG

derived from plasmid RK2

6,540 6,560 6,580 6,600 6,620

TGGGGTCAGTTCCGGCTGGGGGTTCAGCAGCCAGCGCTTTACTGAGATCCTCTTCCGCTTCCTCGCTCACTGACTCGCTGCGCTCGGTCGTTCGGCTGCGGCGAGCG

ColE1

6,640 6,660 6,680 6,700 6,720 6,740

GTATCAGCTCACTCAAAGGCGGTAATACGGTTATCCACAGAATCAGGGGATAACGCAGGAAAGAACATGTGAGCAAAAGGCCAGCAAAAGGCCAGGAACCGTAAAAA

ColE1

6,760 6,780 6,800 6,820 6,840

GGCCGCGTTGCTGGCGTTTTTCCATAGGCTCCGCCCCCCTGACGAGCATCACAAAAATCGACGCTCAAGTCAGAGGTGGCGAAACCCGACAGGACTATAAAGATACC

ColE1

6,860 6,880 6,900 6,920 6,940

AGGCGTTTCCCCCTGGAAGCTCCCTCGTGCGCTCTCCTGTTCCGACCCTGCCGCTTACCGGATACCTGTCCGCCTTTCTCCCTTCGGGAAGCGTGGCGCTTTCTCAT

ColE1

6,960 6,980 7,000 7,020 7,040 7,060

AGCTCACGCTGTAGGTATCTCAGTTCGGTGTAGGTCGTTCGCTCCAAGCTGGGCTGTGTGCACGAACCCCCCGTTCAGCCCGACCGCTGCGCCTTATCCGGTAACTA

ColE1

7,080 7,100 7,120 7,140 7,160

TCGTCTTGAGTCCAACCCGGTAAGACACGACTTATCGCCACTGGCAGCAGCCACTGGTAACAGGATTAGCAGAGCGAGGTATGTAGGCGGTGCTACAGAGTTCTTGA

ColE1

7,180 7,200 7,220 7,240 7,260

AGTGGTGGCCTAACTACGGCTACACTAGAAGAACAGTATTTGGTATCTGCGCTCTGCTGAAGCCAGTTACCTTCGGAAAAAGAGTTGGTAGCTCTTGATCCGGCAAA

ColE1

7,280 7,300 7,320 7,340 7,360 7,380

CAAACCACCGCTGGTAGCGGTGGTTTTTTTGTTTGCAAGCAGCAGATTACGCGCAGAAAAAAAGGATCTCAAGAAGATCCTTTGATCTTTTCTACGGGGTCTGACGC

ColE1

7,400 7,420 7,440 7,460 7,480

TCAGTGGAACGAAAACTCACGTTAAGGGATTTTGGTCATGAGATTATCAAAAAGGATCTTCACCTAGATCCTTTTGGATC

ColE1

7,500 7,520 7,540 7,560

(from 1-1070 bp)

pOIL386: Nb-optimized VpOleosinU1 in pJP3343

TCCTGTGGTTGGCATGCACATACAAATGGACGAACGGATAAACCTTTTCACGCCCTTTTAAATATCCGATTATTCTAATAAACGCTCTTTTCTCTTAGGTTTACCCG

T-DNA right border

20 40 60 80 100

CCAATATATCCTGTCAAACACTGATAGTTTAAACTGAAGGCGGGAAACGACAATCTGCTAGTGGATCTCCCAGTCACGACGTTGTAAAACGGGCGCCCCGCGGAAAG

T-DNA right border

120 140 160 180 200

CTTGCTAGCCAATTGGGGCCCAACGTTCTCGAGTTTTTCTAGAAGGCCTTGGATCCCATGGAGTCAAAGATTCAAATAGAGGACCTAACAGAACTCGCCGTAAAGAC

Pro_35Sx2

220 240 260 280 300 320

TGGCGAACAGTTCATACAGAGTCTCTTACGACTCAATGACAAGAAGAAAATCTTCGTCAACATGGTGGAGCACGACACACTTGTCTACTCCAAAAATATCAAAGATA

Pro_35Sx2

340 360 380 400 420

CAGTCTCAGAAGACCAAAGGGCAATTGAGACTTTTCAACAAAGGGTAATATCCGGAAACCTCCTCGGATTCCATTGCCCAGCTATCTGTCACTTTATTGTGAAGATA

Pro_35Sx2

440 460 480 500 520

GTGGAAAAGGAAGGTGGCTCCTACAAATGCCATCATTGCGATAAAGGAAAGGCCATCGTTGAAGATGCCTCTGCCGACAGTGGTCCCAAAGATGGACCCCCACCCAC

Pro_35Sx2

540 560 580 600 620 640

GAGGAGCATCGTGGAAAAAGAAGACGTTCCAACCACGTCTTCAAAGCAAGTGGATTGATGTGATATCTCCACTGACGTAAGGGATGACGCACAATCCCACTATCCTT

Pro_35Sx2

660 680 700 720 740

CGCAAGACCCTTCCTCTATATAAGGAAGTTCATTTCATTTGGAGAGAACACGGGGGACTGAATTCATGGCTGATTCTCAAGCTGGTCCTAGGGTGCCAGTGGTGAAT

Pro_35Sx2 VpOleosinU1 (opt)

760 780 800 820 840

GGTAATGGTGGTATTGGTGGTTGCCACGCTCTGCTTAGAAGGATTCATCACCACAGCCCTAACAGCTCTCAGGTGGTGGGTTTTCTTACCCTTGCTCTGTCTGGTGC

VpOleosinU1 (opt)

860 880 900 920 940 960

TATTCTGCTTCTGCTGATTGGTATCACCGTGATCGGTGCTACCCTGGGTTTTATTGCTTTCGTGCCTCTGCTGCTGATGACCTCTCCTGTTTGGGTTCCACTTGTGT

VpOleosinU1 (opt)

980 1,000 1,020 1,040 1,060

pOIL386: Nb-optimized VpOleosinU1 in pJP3343 (7528 bp) (from 1071-2...

CCTCTGCTTTCGTTCTTCTTGCTGGTGCTGCTGCTTTCTCTGGTACTGCTGTTGCTACTGCTGCTGGTGTGACTTGGCTGTACAGATACTTCACTGGTAGGCACCCT

VpOleosinU1 (opt)

1,080 1,100 1,120 1,140 1,160

GTGGGTAGCGATAGAGTTGATTATGCTAGGTCCAGGATCGCTGATACCGCTTCTCATGTGAAGGATTACGCTAGGGAATACGGTGGTTACATCCAGAGCAGGATCAA

VpOleosinU1 (opt)

1,180 1,200 1,220 1,240 1,260 1,280

GGATGCTGCTCCTGGTGCTTAGACTAGTGATATCCCTGTGTGAAATTGTTATCCGCTAGCGTGATCGTTCAAACATTTGGCAATAAAGTTTCTTAAGATTGAATCCT

VpOleosinU1 (opt) NOS terminator

1,300 1,320 1,340 1,360 1,380

GTTGCCGGTCTTGCGATGATTATCATATAATTTCTGTTGAATTACGTTAAGCATGTAATAATTAACATGTAATGCATGACGTTATTTATGAGATGGGTTTTTATGAT

NOS terminator

1,400 1,420 1,440 1,460 1,480

TAGAGTCCCGCAATTATACATTTAATACGCGATAGAAAACAAAATATAGCGCGCAAACTAGGATAAATTATCGCGCGCGGTGTCATCTATGTTACTAGATCCCATGG

NOS terminator

1,500 1,520 1,540 1,560 1,580 1,600

GAAGTTCCTATTCCGAAGTTCCTATTCTCTGAAAAGTATAGGAACTTCAGCGATCGCAGACGTCGGGATCTTCTGCAAGCATCTCTATTTCCTGAAGGTCTAACCTC

FRT site enTCUP2 promoter

1,620 1,640 1,660 1,680 1,700

GAAGATTTAAGATTTAATTACGTTTATAATTACAAAATTGATTCTAGTATCTTTAATTTAATGCTTATACATTATTAATTAATTTAGTACTTTCAATTTGTTTTCAG

enTCUP2 promoter

1,720 1,740 1,760 1,780 1,800

AAATTATTTTACTATTTTTTATAAAATAAAAGGGAGAAAATGGCTATTTAAATACTAGCCTATTTTATTTCAATTTTAGCTTAAAATCAGCCCCAATTAGCCCCAAT

enTCUP2 promoter

1,820 1,840 1,860 1,880 1,900 1,920

TTCAAATTCAAATGGTCCAGCCCAATTCCTAAATAACCCACCCCTAACCCGCCCGGTTTCCCCTTTTGATCCATGCAGTCAACGCCCAGAATTTCCCTATATAATTT

enTCUP2 promoter

1,940 1,960 1,980 2,000 2,020

TTTAATTCCCAAACACCCCTAACTCTATCCCATTTCTCACCAACCGCCACATAGATCTATCCTCTTATCTCTCAAACTCTCTCGAACCTTCCCCTAACCCTAGCAGC

enTCUP2 promoter

2,040 2,060 2,080 2,100 2,120 2,140

pOIL386: Nb-optimized VpOleosinU1 in pJP3343 (7528 bp) (from 2141-3...

CTCTCATCATCCTCACCTCAAAACCCACCGGGGCCGGCCATGATTGAACAAGATGGATTGCACGCAGGTTCTCCGGCCGCTTGGGTGGAGAGGCTATTCGGCTATGA

enTCUP2 promoter | neomycin phosphotransferase II

2,160 2,180 2,200 2,220 2,240

CTGGGCACAACAGACAATCGGCTGCTCTGATGCCGCCGTGTTCCGGCTGTCAGCGCAGGGGAGGCCGGTTCTTTTTGTCAAGACCGACCTGTCCGGTGCCCTGAATG

neomycin phosphotransferase II

2,260 2,280 2,300 2,320 2,340

AACTTCAAGACGAGGCAGCGCGGCTATCGTGGCTGGCCACGACGGGCGTTCCTTGCGCAGCTGTGCTCGACGTTGTCACTGAAGCGGGAAGGGACTGGCTGCTATTG

neomycin phosphotransferase II

2,360 2,380 2,400 2,420 2,440 2,460

GGCGAAGTGCCGGGGCAGGATCTCCTGTCATCTCACCTTGCTCCTGCCGAGAAAGTATCCATCATGGCTGATGCAATGCGGCGGCTGCATACGCTTGATCCGGCTAC

neomycin phosphotransferase II

2,480 2,500 2,520 2,540 2,560

CTGCCCATTCGACCACCAAGCGAAACATCGCATCGAGCGAGCACGTACTCGGATGGAAGCCGGTCTTGTCGATCAGGATGATCTGGACGAAGAGCATCAGGGGCTCG

neomycin phosphotransferase II

2,580 2,600 2,620 2,640 2,660

CGCCAGCCGAACTGTTCGCCAGGCTCAAGGCGCGCATGCCCGACGGCGAGGATCTCGTCGTGACTCATGGCGATGCCTGCTTGCCGAATATCATGGTGGAAAATGGC

neomycin phosphotransferase II

2,680 2,700 2,720 2,740 2,760 2,780

CGCTTTTCTGGATTCATCGACTGTGGCCGGCTGGGTGTGGCGGACCGCTATCAGGACATAGCGTTGGCTACCCGTGATATTGCTGAAGAGCTTGGCGGCGAATGGGC

neomycin phosphotransferase II

2,800 2,820 2,840 2,860 2,880

TGACCGCTTCCTCGTGCTTTACGGTATCGCCGCTCCCGATTCGCAGCGCATCGCCTTCTATCGCCTTCTTGACGAGTTCTTCTGAGGCGCGCCGATCGTTCAAACAT

neomycin phosphotransferase II | NOS ...tor

2,900 2,920 2,940 2,960 2,980

TTGGCAATAAAGTTTCTTAAGATTGAATCCTGTTGCCGGTCTTGCGATGATTATCATATAATTTCTGTTGAATTACGTTAAGCATGTAATAATTAACATGTAATGCA

NOS terminator

3,000 3,020 3,040 3,060 3,080 3,100

TGACGTTATTTATGAGATGGGTTTTTATGATTAGAGTCCCGCAATTATACATTTAATACGCGATAGAAAACAAAATATAGCGCGCAAACTAGGATAAATTATCGCGC

NOS terminator

3,120 3,140 3,160 3,180 3,200

pOIL386: Nb-optimized VpOleosinU1 in pJP3343 (7528 bp) (from 3211-4...

GCGGTGTCATCTATGTTACTAGATCCCTAGGGAAGTTCCTATTCCGAAGTTCCTATTCTCTGAAAAGTATAGGAACTTCTTTGCGTATTGGGCGCTCTTGGCCTTTT

NOS terminator | FRT site

3,220 3,240 3,260 3,280 3,300

TGGCCACCGGTCGTACGGTTAAAACCACCCCAGTACATTAAAAACGTCCGCAATGTGTTATTAAGTTGTCTAAGCGTCAATTTGTTTACACCACAATATATCCTGCC

T-DNA left border

3,320 3,340 3,360 3,380 3,400 3,420

ACCAGCCAGCCAACAGCTCCCCGACCGGCAGCTCGGCACAAAATCACCACTCGATACAGGCAGCCCATCAGTCCACTAGACGCTCACCGGGCTGGTTGCCCTCGCCG

T-DNA left border | RK2 oriV

3,440 3,460 3,480 3,500 3,520

CTGGGCTGGCGGCCGTCTATGGCCCTGCAAACGCGCCAGAAACGCCGTCGAAGCCGTGTGCGAGACACCGCAGCCGCCGGCGTTGTGGATACCTCGCGGAAAACTTG

RK2 oriV

3,540 3,560 3,580 3,600 3,620

GCCCTCACTGACAGATGAGGGGCGGACGTTGACACTTGAGGGGCCGACTCACCCGGCGCGGCGTTGACAGATGAGGGGCAGGCTCGATTTCGGCCGGCGACGTGGAG

RK2 oriV

3,640 3,660 3,680 3,700 3,720 3,740

CTGGCCAGCCTCGCAAATCGGCGAAAACGCCTGATTTTACGCGAGTTTCCCACAGATGATGTGGACAAGCCTGGGGATAAGTGCCCTGCGGTATTGACACTTGAGGG

RK2 oriV

3,760 3,780 3,800 3,820 3,840

GCGCGACTACTGACAGATGAGGGGCGCGATCCTTGACACTTGAGGGGCAGAGTGCTGACAGATGAGGGGCGCACCTATTGACATTTGAGGGGCTGTCCACAGGCAGA

RK2 oriV

3,860 3,880 3,900 3,920 3,940

AAATCCAGCATTTGCAAGGGTTTCCGCCCGTTTTTCGGCCACCGCTAACCTGTCTTTTAACCTGCTTTTAAACCAATATTTATAAACCTTGTTTTTAACCAGGGCTG

RK2 oriV

3,960 3,980 4,000 4,020 4,040 4,060

CGCCCTGTGCGCGTGACCGCGCACGCCGAAGGGGGGTGCCCCCCCTTCTCGAACCCTCCCGGCCCGCTCTCGCGTTGGCAGCATCACCCATAATTGTGGTTTCAAAA

RK2 oriV

4,080 4,100 4,120 4,140 4,160

TCGGCTCCGTCGATACTATGTTATACGCCAACTTTGAAAACAACTTTGAAAAAGCTGTTTTCTGGTATTTAAGGTTTTAGAATGCAAGGAACAGTGAATTGGAGTTC

4,180 4,200 4,220 4,240 4,260 4,280

GTCTTGTTATAATTAGCTTCTTGGGGTATTTAAATACTGTAGAAAAGAGGAAGGAAATAATAAATGGCTAAAATGAGAATATCACCGGAATTGAAAAAACTGATCGA

neomycin-kanamycin ...ansferase type III

4,300 4,320 4,340 4,360 4,380

AAAATACCGCTGCGTAAAAGATACGGAAGGAATGTCTCCTGCTAAGGTATATAAGCTGGTGGGAGAAAATGAAAACCTATATTTAAAAATGACGGACAGCCGGTATA

neomycin-kanamycin phosphotransferase type III

4,400 4,420 4,440 4,460 4,480

AAGGGACCACCTATGATGTGGAACGGGAAAAGGACATGATGCTATGGCTGGAAGGAAAGCTGCCTGTTCCAAAGGTCCTGCACCTTGAACGGCATGATGGCTGGAGC

neomycin-kanamycin phosphotransferase type III

4,500 4,520 4,540 4,560 4,580 4,600

AATCTGCTCATGAGTGAGGCCGATGGCGTCCTTTGCTCGGAAGAGTATGAAGATGAACAAAGCCCTGAAAAGATTATCGAGCTGTATGCGGAGTGCATCAGGCTCTT

neomycin-kanamycin phosphotransferase type III

4,620 4,640 4,660 4,680 4,700

TCACTCCATCGACATATCGGATTGTCCCTATACGAATAGCTTAGACAGCCGCTTAGCCGAATTGGATTACTTACTGAATAACGATCTGGCCGATGTGGATTGCGAAA

neomycin-kanamycin phosphotransferase type III

4,720 4,740 4,760 4,780 4,800

ACTGGGAAGAAGACACCCCATTTAAAGATCCGCGCGAGCTGTATGATTTTTTAAAGACGGAAAAGCCCGAAGAGGAACTTGTCTTTTCCCACGGCGACCTGGGAGAC

neomycin-kanamycin phosphotransferase type III

4,820 4,840 4,860 4,880 4,900 4,920

AGCAACATCTTTGTGAAAGATGGCAAAGTAAGTGGCTTTATTGATCTTGGGAGAAGCGGCAGGGCGGACAAGTGGTATGACATTGCCTTCTGCGTCCGGTCGATCAG

neomycin-kanamycin phosphotransferase type III

4,940 4,960 4,980 5,000 5,020

GGAGGATATTGGGGAAGAACAGTATGTCGAGCTATTTTTTGACTTACTGGGGATCAAGCCTGATTGGGAGAAAATAAAATATTATATTTTACTGGATGAATTGTTTT

neomycin-kanamycin phosphotransferase type III

5,040 5,060 5,080 5,100 5,120

AGTACCTAGATGTGGCGCAACGATGCTGGCGACAAGCAGGAGCGCACCGACTTCTTCCGCATCAAGTGTTTTGGCTCTCAGGCCGAGGCCCACGGCAAGTATTTGGG

5,140 5,160 5,180 5,200 5,220 5,240

CAAGGGGTCGCTGGTATTCGTGCAGGGCAAGATTCGGAATACCAAGTACGAGAAGGACGGCCAGACGGTCTACGGGACCGACTTCATTGCCGATAAGGTGGATTATC

5,260 5,280 5,300 5,320 5,340

TGGACACCAAGGCACCAGGCGGATCAAATCAGGAATAAGGGCACATTGCCCCGGCGTGAGTCGGGGCAATCCCGCAAGGAGGGTGAATGAATCGGACGTTTGACCGG

derived...id RK2

5,360 5,380 5,400 5,420 5,440

AAGGCATACAGGCAAGAACTGATCGACGCGGGGTTTTCCGCCGAGGATGCCGAAACCATCGCAAGCCGCACCGTCATGCGTGCGCCCCGCGAAACCTTCCAGTCCGT

derived from plasmid RK2

5,460 5,480 5,500 5,520 5,540 5,560

CGGCTCGATGGCCCAGCAAGCTACGGCCAAGATCGAGCGCGACAGCGTGCAACTGGCTCCCCCTGCCCTGCCCGCGCCATCGGCCGCCGTGGAGCGTTCGCGTCGTC

derived from plasmid RK2

5,580 5,600 5,620 5,640 5,660

TCGAACAGGAGGCGGCAGGTTTGGCGAAGTCGATGACCATCGACACGCGAGGAACTATGACGACCAAGAAGCGAAAAACCGCCGGCGAGGACCTGGCAAAACAGGTC

derived from plasmid RK2

5,680 5,700 5,720 5,740 5,760

AGCGAGGCCAAGCAAGCCGCGTTGCTGAAACACACGAAGCAGCAGATCAAGGAAATGCAGCTTTCCTTGTTCGATATTGCGCCGTGGCCGGACACGATGCGAGCGAT

derived from plasmid RK2

5,780 5,800 5,820 5,840 5,860 5,880

GCCAAACGACACGGCCCGCTCTGCCCTGTTCACCACGCGCAACAAGAAAATCCCGCGCGAGGCGCTGCAAAACAAGGTCATTTTCCACGTCAACAAGGACGTGAAGA

derived from plasmid RK2

5,900 5,920 5,940 5,960 5,980

TCACCTACACCGGCGTCGAGCTGCGGGCCGACGATGACGAACTGGTGTGGCAGCAGGTGTTGGAGTACGCGAAGCGCACCCCTATCGGCGAGCCGATCACCTTCACG

derived from plasmid RK2

6,000 6,020 6,040 6,060 6,080

TTCTACGAGCTTTGCCAGGACCTGGGCTGGTCGATCAATGGCCGGTATTACACGAAGGCCGAGGAATGCCTGTCGCGCCTACAGGCGACGGCGATGGGCTTCACGTC

derived from plasmid RK2

6,100 6,120 6,140 6,160 6,180 6,200

CGACCGCGTTGGGCACCTGGAATCGGTGTCGCTGCTGCACCGCTTCCGCGTCCTGGACCGTGGCAAGAAAACGTCCCGTTGCCAGGTCCTGATCGACGAGGAAATCG

derived from plasmid RK2

6,220 6,240 6,260 6,280 6,300

TCGTGCTGTTTGCTGGCGACCACTACACGAAATTCATATGGGAGAAGTACCGCAAGCTGTCGCCGACGGCCCGACGGATGTTCGACTATTTCAGCTCGCACCGGGAG

derived from plasmid RK2

6,320 6,340 6,360 6,380 6,400 6,420

pOIL386: Nb-optimized VpOleosinU1 in pJP3343 (7528 bp) (from 6421-7...

CCGTACCCGCTCAAGCTGGAAACCTTCCGCCTCATGTGCGGATCGGATTCCACCCGCGTGAAGAAGTGGCGCGAGCAGGTCGGCGAAGCCTGCGAAGAGTTGCGAGG

derived from plasmid RK2

6,440 6,460 6,480 6,500 6,520

CAGCGGCCTGGTGGAACACGCCTGGGTCAATGATGACCTGGTGCATTGCAAACGCTAGGGCCTTGTGGGGTCAGTTCCGGCTGGGGGTTCAGCAGCCAGCGCTTTAC

derived from plasmid RK2

6,540 6,560 6,580 6,600 6,620

TGAGATCCTCTTCCGCTTCCTCGCTCACTGACTCGCTGCGCTCGGTCGTTCGGCTGCGGCGAGCGGTATCAGCTCACTCAAAGGCGGTAATACGGTTATCCACAGAA

ColE1

6,640 6,660 6,680 6,700 6,720 6,740

TCAGGGGATAACGCAGGAAAGAACATGTGAGCAAAAGGCCAGCAAAAGGCCAGGAACCGTAAAAAGGCCGCGTTGCTGGCGTTTTTCCATAGGCTCCGCCCCCCTGA

ColE1

6,760 6,780 6,800 6,820 6,840

CGAGCATCACAAAAATCGACGCTCAAGTCAGAGGTGGCGAAACCCGACAGGACTATAAAGATACCAGGCGTTTCCCCCTGGAAGCTCCCTCGTGCGCTCTCCTGTTC

ColE1

6,860 6,880 6,900 6,920 6,940

CGACCCTGCCGCTTACCGGATACCTGTCCGCCTTTCTCCCTTCGGGAAGCGTGGCGCTTTCTCATAGCTCACGCTGTAGGTATCTCAGTTCGGTGTAGGTCGTTCGC

ColE1

6,960 6,980 7,000 7,020 7,040 7,060

TCCAAGCTGGGCTGTGTGCACGAACCCCCCGTTCAGCCCGACCGCTGCGCCTTATCCGGTAACTATCGTCTTGAGTCCAACCCGGTAAGACACGACTTATCGCCACT

ColE1

7,080 7,100 7,120 7,140 7,160

GGCAGCAGCCACTGGTAACAGGATTAGCAGAGCGAGGTATGTAGGCGGTGCTACAGAGTTCTTGAAGTGGTGGCCTAACTACGGCTACACTAGAAGAACAGTATTTG

ColE1

7,180 7,200 7,220 7,240 7,260

GTATCTGCGCTCTGCTGAAGCCAGTTACCTTCGGAAAAAGAGTTGGTAGCTCTTGATCCGGCAAACAAACCACCGCTGGTAGCGGTGGTTTTTTTGTTTGCAAGCAG

ColE1

7,280 7,300 7,320 7,340 7,360 7,380

CAGATTACGCGCAGAAAAAAAGGATCTCAAGAAGATCCTTTGATCTTTTCTACGGGGTCTGACGCTCAGTGGAACGAAAACTCACGTTAAGGGATTTTGGTCATGAG

ColE1

7,400 7,420 7,440 7,460 7,480

pOIL386: Nb-optimized VpOleosinU1 in pJP3343 (7528 bp) (from 7491-7...

ATTATCAAAAAGGATCTTCACCTAGATCCTTTTGGATC

ColE1

7,500 7,510 7,520

(from 1-1070 bp)

pOIL387: Nb-optimized PaOleosinM in pJP3343

TCCTGTGGTTGGCATGCACATACAAATGGACGAACGGATAAACCTTTTCACGCCCTTTTAAATATCCGATTATTCTAATAAACGCTCTTTTCTCTTAGGTTTACCCG

T-DNA right border

20 40 60 80 100

CCAATATATCCTGTCAAACACTGATAGTTTAAACTGAAGGCGGGAAACGACAATCTGCTAGTGGATCTCCCAGTCACGACGTTGTAAAACGGGCGCCCCGCGGAAAG

T-DNA right border

120 140 160 180 200

CTTGCTAGCCAATTGGGGCCCAACGTTCTCGAGTTTTTCTAGAAGGCCTTGGATCCCATGGAGTCAAAGATTCAAATAGAGGACCTAACAGAACTCGCCGTAAAGAC

Pro_35Sx2

220 240 260 280 300 320

TGGCGAACAGTTCATACAGAGTCTCTTACGACTCAATGACAAGAAGAAAATCTTCGTCAACATGGTGGAGCACGACACACTTGTCTACTCCAAAAATATCAAAGATA

Pro_35Sx2

340 360 380 400 420

CAGTCTCAGAAGACCAAAGGGCAATTGAGACTTTTCAACAAAGGGTAATATCCGGAAACCTCCTCGGATTCCATTGCCCAGCTATCTGTCACTTTATTGTGAAGATA

Pro_35Sx2

440 460 480 500 520

GTGGAAAAGGAAGGTGGCTCCTACAAATGCCATCATTGCGATAAAGGAAAGGCCATCGTTGAAGATGCCTCTGCCGACAGTGGTCCCAAAGATGGACCCCCACCCAC

Pro_35Sx2

540 560 580 600 620 640

GAGGAGCATCGTGGAAAAAGAAGACGTTCCAACCACGTCTTCAAAGCAAGTGGATTGATGTGATATCTCCACTGACGTAAGGGATGACGCACAATCCCACTATCCTT

Pro_35Sx2

660 680 700 720 740

CGCAAGACCCTTCCTCTATATAAGGAAGTTCATTTCATTTGGAGAGAACACGGGGGACTGAATTCATGGCTGATCAACCTAAGACCATCAAGCAGACTGAGAGGGCT

Pro_35Sx2 | | PaOleosinM (opt)

760 780 800 820 840

CCTAATACCAACCAGACCGCTATTTTCCTTACCGCTGCTACTGTGGGTGGAACCCTTCTTGTTCTTTCTGGTCTTACCTTGACCGCTACCGCTATCTCTCTTCTTAT

PaOleosinM (opt)

860 880 900 920 940 960

GGCTACCCCTCTTCTGGTGATCTGGTCCCCTATTCTTATGCCAACCACCTTCCTTGTGGCTACCGGTTTCATCTTCAGCAGCGGTTTTGGTATCGCTGCTCTTTCTG

PaOleosinM (opt)

980 1,000 1,020 1,040 1,060

pOIL387: Nb-optimized PaOleosinM in pJP3343 (7516 bp) (from 1071-21...

TTGTGGCTTGGATGTACAGGTACGCTACTGGTAAACACCCTCCTGGTTCTGAGCAGCTGGATCATGTTATGAAGGTGTTGGTGCACGAGGCTAAGATCATGAAGAAC
PaOleosinM (opt)
1,080 1,100 1,120 1,140 1,160

AAGGCTAAAGAGTACGCTTACCAGGTTAGGGATATGGCTCAGCAGATTACCAAGAGGGTGCAGAACATGGGAAACAGCGAGATCCTGTTCAGCGAGAACAGGCTTAA
PaOleosinM (opt)
1,180 1,200 1,220 1,240 1,260 1,280

GGTGTTCTAGACTAGTGATATCCCTGTGTGAAATTGTTATCCGCTAGCGTGATCGTTCAAACATTTGGCAATAAAGTTTCTTAAGATTGAATCCTGTTGCCGGTCTT
Pa...) NOS terminator
1,300 1,320 1,340 1,360 1,380

GCGATGATTATCATATAATTTCTGTTGAATTACGTTAAGCATGTAATAATTAACATGTAATGCATGACGTTATTTATGAGATGGGTTTTTATGATTAGAGTCCCGCA
NOS terminator
1,400 1,420 1,440 1,460 1,480

ATTATACATTTAATACGCGATAGAAAACAAAATATAGCGCGCAAACTAGGATAAATTATCGCGCGCGGTGTCATCTATGTTACTAGATCCCATGGGAAGTTCCTATT
NOS terminator FRT...te
1,500 1,520 1,540 1,560 1,580 1,600

CCGAAGTTCCTATTCTCTGAAAAGTATAGGAACTTCAGCGATCGCAGACGTCGGGATCTTCTGCAAGCATCTCTATTTCCTGAAGGTCTAACCTCGAAGATTTAAGA
FRT site enTCUP2 promoter
1,620 1,640 1,660 1,680 1,700

TTTAATTACGTTTATAATTACAAAATTGATTCTAGTATCTTTAATTTAATGCTTATACATTATTAATTAATTTAGTACTTTCAATTTGTTTTCAGAAATTATTTTAC
enTCUP2 promoter
1,720 1,740 1,760 1,780 1,800

TATTTTTTATAAAATAAAAGGGAGAAAATGGCTATTTAAATACTAGCCTATTTTATTTCAATTTTAGCTTAAAATCAGCCCCAATTAGCCCCAATTTCAAATTCAAA
enTCUP2 promoter
1,820 1,840 1,860 1,880 1,900 1,920

TGGTCCAGCCCAATTCCTAAATAACCCACCCCTAACCCGCCCGGTTTCCCCTTTTGATCCATGCAGTCAACGCCCAGAATTTCCCTATATAATTTTTTAATTCCCAA
enTCUP2 promoter
1,940 1,960 1,980 2,000 2,020

ACACCCCTAACTCTATCCCATTTCTCACCAACCGCCACATAGATCTATCCTCTTATCTCTCAAACTCTCTCGAACCTTCCCCTAACCCTAGCAGCCTCTCATCATCC
enTCUP2 promoter
2,040 2,060 2,080 2,100 2,120 2,140

pOIL387: Nb-optimized PaOleosinM in pJP3343 (7516 bp) (from 2141-32...

TCACCTCAAAACCCACCGGGGCCGGCCATGATTGAACAAGATGGATTGCACGCAGGTTCTCCGGCCGCTTGGGTGGAGAGGCTATTCGGCTATGACTGGGCACAACA

enTCUP...moter neomycin phosphotransferase II

2,160 2,180 2,200 2,220 2,240

GACAATCGGCTGCTCTGATGCCGCCGTGTTCCGGCTGTCAGCGCAGGGGAGGCCGGTTCTTTTTGTCAAGACCGACCTGTCCGGTGCCCTGAATGAACTTCAAGACG

neomycin phosphotransferase II

2,260 2,280 2,300 2,320 2,340

AGGCAGCGCGGCTATCGTGGCTGGCCACGACGGGCGTTCCTTGCGCAGCTGTGCTCGACGTTGTCACTGAAGCGGGAAGGGACTGGCTGCTATTGGGCGAAGTGCCG

neomycin phosphotransferase II

2,360 2,380 2,400 2,420 2,440 2,460

GGGCAGGATCTCCTGTCATCTCACCTTGCTCCTGCCGAGAAAGTATCCATCATGGCTGATGCAATGCGGCGGCTGCATACGCTTGATCCGGCTACCTGCCCATTCGA

neomycin phosphotransferase II

2,480 2,500 2,520 2,540 2,560

CCACCAAGCGAAACATCGCATCGAGCGAGCACGTACTCGGATGGAAGCCGGTCTTGTCGATCAGGATGATCTGGACGAAGAGCATCAGGGGCTCGCGCCAGCCGAAC

neomycin phosphotransferase II

2,580 2,600 2,620 2,640 2,660

TGTTCGCCAGGCTCAAGGCGCGCATGCCCGACGGCGAGGATCTCGTCGTGACTCATGGCGATGCCTGCTTGCCGAATATCATGGTGGAAAATGGCCGCTTTTCTGGA

neomycin phosphotransferase II

2,680 2,700 2,720 2,740 2,760 2,780

TTCATCGACTGTGGCCGGCTGGGTGTGGCGGACCGCTATCAGGACATAGCGTTGGCTACCCGTGATATTGCTGAAGAGCTTGGCGGCGAATGGGCTGACCGCTTCCT

neomycin phosphotransferase II

2,800 2,820 2,840 2,860 2,880

CGTGCTTTACGGTATCGCCGCTCCCGATTCGCAGCGCATCGCCTTCTATCGCCTTCTTGACGAGTTCTTCTGAGGCGCGCCGATCGTTCAAACATTTGGCAATAAAG

neomycin phosphotransferase II NOS terminator

2,900 2,920 2,940 2,960 2,980

TTTCTTAAGATTGAATCCTGTTGCCGGTCTTGCGATGATTATCATATAATTTCTGTTGAATTACGTTAAGCATGTAATAATTAACATGTAATGCATGACGTTATTTA

NOS terminator

3,000 3,020 3,040 3,060 3,080 3,100

TGAGATGGGTTTTTATGATTAGAGTCCCGCAATTATACATTTAATACGCGATAGAAAACAAAATATAGCGCGCAAACTAGGATAAATTATCGCGCGCGGTGTCATCT

NOS terminator

3,120 3,140 3,160 3,180 3,200

pOIL387: Nb-optimized PaOleosinM in pJP3343 (7516 bp) (from 3211-42...

ATGTTACTAGATCCCTAGGGAAGTTCCTATTCCGAAGTTCCTATTCTCTGAAAAGTATAGGAACTTCTTTGCGTATTGGGCGCTCTTGGCCTTTTTGGCCACCGGTC

NOS...or FRT site

3,220 3,240 3,260 3,280 3,300

GTACGGTTAAAACCACCCCAGTACATTAAAAACGTCCGCAATGTGTTATTAAGTTGTCTAAGCGTCAATTTGTTTACACCACAATATATCCTGCCACCAGCCAGCCA

T-DNA left border

3,320 3,340 3,360 3,380 3,400 3,420

ACAGCTCCCCGACCGGCAGCTCGGCACAAAATCACCACTCGATACAGGCAGCCCATCAGTCCACTAGACGCTCACCGGGCTGGTTGCCCTCGCCGCTGGGCTGGCGG

T-DNA left border RK2 oriV

3,440 3,460 3,480 3,500 3,520

CCGTCTATGGCCCTGCAAACGCGCCAGAAACGCCGTCGAAGCCGTGTGCGAGACACCGCAGCCGCCGGCGTTGTGGATACCTCGCGGAAAACTTGGCCCTCACTGAC

RK2 oriV

3,540 3,560 3,580 3,600 3,620

AGATGAGGGGCGGACGTTGACACTTGAGGGGCCGACTCACCCGGCGCGGCGTTGACAGATGAGGGGCAGGCTCGATTTCGGCCGGCGACGTGGAGCTGGCCAGCCTC

RK2 oriV

3,640 3,660 3,680 3,700 3,720 3,740

GCAAATCGGCGAAAACGCCTGATTTTACGCGAGTTTCCCACAGATGATGTGGACAAGCCTGGGGATAAGTGCCCTGCGGTATTGACACTTGAGGGGCGCGACTACTG

RK2 oriV

3,760 3,780 3,800 3,820 3,840

ACAGATGAGGGGCGCGATCCTTGACACTTGAGGGGCAGAGTGCTGACAGATGAGGGGCGCACCTATTGACATTTGAGGGGCTGTCCACAGGCAGAAAATCCAGCATT

RK2 oriV

3,860 3,880 3,900 3,920 3,940

TGCAAGGGTTTCCGCCCGTTTTTCGGCCACCGCTAACCTGTCTTTTAACCTGCTTTTAAACCAATATTTATAAACCTTGTTTTTAACCAGGGCTGCGCCCTGTGCGC

RK2 oriV

3,960 3,980 4,000 4,020 4,040 4,060

GTGACCGCGCACGCCGAAGGGGGGTGCCCCCCCTTCTCGAACCCTCCCGGCCCGCTCTCGCGTTGGCAGCATCACCCATAATTGTGGTTTCAAAATCGGCTCCGTCG

RK2 oriV

4,080 4,100 4,120 4,140 4,160

ATACTATGTTATACGCCAACTTTGAAAACAACTTTGAAAAAGCTGTTTTCTGGTATTTAAGGTTTTAGAATGCAAGGAACAGTGAATTGGAGTTCGTCTTGTTATAA

4,180 4,200 4,220 4,240 4,260 4,280

TTAGCTTCTTGGGGTATTTAAATACTGTAGAAAAGAGGAAGGAAATAATAAATGGCTAAAATGAGAATATCACCGGAATTGAAAAAACTGATCGAAAAATACCGCTG
neomycin-kanamycin phosphotransferase type III
4,300 4,320 4,340 4,360 4,380

CGTAAAAGATACGGAAGGAATGTCTCCTGCTAAGGTATATAAGCTGGTGGGAGAAAATGAAAACCTATATTTAAAAATGACGGACAGCCGGTATAAAGGGACCACCT
neomycin-kanamycin phosphotransferase type III
4,400 4,420 4,440 4,460 4,480

ATGATGTGGAACGGGAAAAGGACATGATGCTATGGCTGGAAGGAAAGCTGCCTGTTCCAAAGGTCCTGCACCTTGAACGGCATGATGGCTGGAGCAATCTGCTCATG
neomycin-kanamycin phosphotransferase type III
4,500 4,520 4,540 4,560 4,580 4,600

AGTGAGGCCGATGGCGTCCTTTGCTCGGAAGAGTATGAAGATGAACAAAGCCCTGAAAAGATTATCGAGCTGTATGCGGAGTGCATCAGGCTCTTTCACTCCATCGA
neomycin-kanamycin phosphotransferase type III
4,620 4,640 4,660 4,680 4,700

CATATCGGATTGTCCCTATACGAATAGCTTAGACAGCCGCTTAGCCGAATTGGATTACTTACTGAATAACGATCTGGCCGATGTGGATTGCGAAAACTGGGAAGAAG
neomycin-kanamycin phosphotransferase type III
4,720 4,740 4,760 4,780 4,800

ACACCCCATTTAAAGATCCGCGCGAGCTGTATGATTTTTTAAAGACGGAAAAGCCCGAAGAGGAACTTGTCTTTTCCCACGGCGACCTGGGAGACAGCAACATCTTT
neomycin-kanamycin phosphotransferase type III
4,820 4,840 4,860 4,880 4,900 4,920

GTGAAAGATGGCAAAGTAAGTGGCTTTATTGATCTTGGGAGAAGCGGCAGGGCGGACAAGTGGTATGACATTGCCTTCTGCGTCCGGTCGATCAGGGAGGATATTGG
neomycin-kanamycin phosphotransferase type III
4,940 4,960 4,980 5,000 5,020

GGAAGAACAGTATGTCGAGCTATTTTTTGACTTACTGGGGATCAAGCCTGATTGGGAGAAAATAAAATATTATATTTTACTGGATGAATTGTTTTAGTACCTAGATG
neomycin-kanamycin phosphotransferase type III
5,040 5,060 5,080 5,100 5,120

TGGCGCAACGATGCTGGCGACAAGCAGGAGCGCACCGACTTCTTCCGCATCAAGTGTTTTGGCTCTCAGGCCGAGGCCCACGGCAAGTATTTGGGCAAGGGGTCGCT
5,140 5,160 5,180 5,200 5,220 5,240

GGTATTCGTGCAGGGCAAGATTCGGAATACCAAGTACGAGAAGGACGGCCAGACGGTCTACGGGACCGACTTCATTGCCGATAAGGTGGATTATCTGGACACCAAGG
5,260 5,280 5,300 5,320 5,340

CACCAGGCGGATCAAATCAGGAATAAGGGCACATTGCCCCGGCGTGAGTCGGGGCAATCCCGCAAGGAGGGTGAATGAATCGGACGTTTGACCGGAAGGCATACAGG
derived from plasmid RK2
5,360 5,380 5,400 5,420 5,440

CAAGAACTGATCGACGCGGGGTTTTCCGCCGAGGATGCCGAAACCATCGCAAGCCGCACCGTCATGCGTGCGCCCCGCGAAACCTTCCAGTCCGTCGGCTCGATGGC

derived from plasmid RK2

5,460 5,480 5,500 5,520 5,540 5,560

CCAGCAAGCTACGGCCAAGATCGAGCGCGACAGCGTGCAACTGGCTCCCCCTGCCCTGCCCGCGCCATCGGCCGCCGTGGAGCGTTCGCGTCGTCTCGAACAGGAGG

derived from plasmid RK2

5,580 5,600 5,620 5,640 5,660

CGGCAGGTTTGGCGAAGTCGATGACCATCGACACGCGAGGAACTATGACGACCAAGAAGCGAAAAACCGCCGGCGAGGACCTGGCAAAACAGGTCAGCGAGGCCAAG

derived from plasmid RK2

5,680 5,700 5,720 5,740 5,760

CAAGCCGCGTTGCTGAAACACACGAAGCAGCAGATCAAGGAAATGCAGCTTTCCTTGTTCGATATTGCGCCGTGGCCGGACACGATGCGAGCGATGCCAAACGACAC

derived from plasmid RK2

5,780 5,800 5,820 5,840 5,860 5,880

GGCCCGCTCTGCCCTGTTCACCACGCGCAACAAGAAAATCCCGCGCGAGGCGCTGCAAAACAAGGTCATTTTCCACGTCAACAAGGACGTGAAGATCACCTACACCG

derived from plasmid RK2

5,900 5,920 5,940 5,960 5,980

GCGTCGAGCTGCGGGCCGACGATGACGAACTGGTGTGGCAGCAGGTGTTGGAGTACGCGAAGCGCACCCCTATCGGCGAGCCGATCACCTTCACGTTCTACGAGCTT

derived from plasmid RK2

6,000 6,020 6,040 6,060 6,080

TGCCAGGACCTGGGCTGGTCGATCAATGGCCGGTATTACACGAAGGCCGAGGAATGCCTGTCGCGCCTACAGGCGACGGCGATGGGCTTCACGTCCGACCGCGTTGG

derived from plasmid RK2

6,100 6,120 6,140 6,160 6,180 6,200

GCACCTGGAATCGGTGTCGCTGCTGCACCGCTTCCGCGTCCTGGACCGTGGCAAGAAAACGTCCCGTTGCCAGGTCCTGATCGACGAGGAAATCGTCGTGCTGTTTG

derived from plasmid RK2

6,220 6,240 6,260 6,280 6,300

CTGGCGACCACTACACGAAATTCATATGGGAGAAGTACCGCAAGCTGTCGCCGACGGCCCGACGGATGTTCGACTATTTCAGCTCGCACCGGGAGCCGTACCCGCTC

derived from plasmid RK2

6,320 6,340 6,360 6,380 6,400 6,420

AAGCTGGAAACCTTCCGCCTCATGTGCGGATCGGATTCCACCCGCGTGAAGAAGTGGCGCGAGCAGGTCGGCGAAGCCTGCGAAGAGTTGCGAGGCAGCGGCCTGGT

derived from plasmid RK2

6,440 6,460 6,480 6,500 6,520

pOIL387: Nb-optimized PaOleosinM in pJP3343 (7516 bp) (from 6528-75...

GGAACACGCCTGGGTCAATGATGACCTGGTGCATTGCAAACGCTAGGGCCTTGTGGGGTCAGTTCCGGCTGGGGGTTCAGCAGCCAGCGCTTTACTGAGATCCTCTT

derived from plasmid RK2

6,540 6,560 6,580 6,600 6,620

CCGCTTCCTCGCTCACTGACTCGCTGCGCTCGGTCGTTCGGCTGCGGCGAGCGGTATCAGCTCACTCAAAGGCGGTAATACGGTTATCCACAGAATCAGGGGATAAC

ColE1

6,640 6,660 6,680 6,700 6,720 6,740

GCAGGAAAGAACATGTGAGCAAAAGGCCAGCAAAAGGCCAGGAACCGTAAAAAGGCCGCGTTGCTGGCGTTTTTCCATAGGCTCCGCCCCCCTGACGAGCATCACAA

ColE1

6,760 6,780 6,800 6,820 6,840

AAATCGACGCTCAAGTCAGAGGTGGCGAAACCCGACAGGACTATAAAGATACCAGGCGTTTCCCCCTGGAAGCTCCCTCGTGCGCTCTCCTGTTCCGACCCTGCCGC

ColE1

6,860 6,880 6,900 6,920 6,940

TTACCGGATACCTGTCCGCCTTTCTCCCTTCGGGAAGCGTGGCGCTTTCTCATAGCTCACGCTGTAGGTATCTCAGTTCGGTGTAGGTCGTTCGCTCCAAGCTGGGC

ColE1

6,960 6,980 7,000 7,020 7,040 7,060

TGTGTGCACGAACCCCCCGTTCAGCCCGACCGCTGCGCCTTATCCGGTAACTATCGTCTTGAGTCCAACCCGGTAAGACACGACTTATCGCCACTGGCAGCAGCCAC

ColE1

7,080 7,100 7,120 7,140 7,160

TGGTAACAGGATTAGCAGAGCGAGGTATGTAGGCGGTGCTACAGAGTTCTTGAAGTGGTGGCCTAACTACGGCTACACTAGAAGAACAGTATTTGGTATCTGCGCTC

ColE1

7,180 7,200 7,220 7,240 7,260

TGCTGAAGCCAGTTACCTTCGGAAAAAGAGTTGGTAGCTCTTGATCCGGCAAACAAACCACCGCTGGTAGCGGTGGTTTTTTTGTTTGCAAGCAGCAGATTACGCGC

ColE1

7,280 7,300 7,320 7,340 7,360 7,380

AGAAAAAAAGGATCTCAAGAAGATCCTTTGATCTTTTCTACGGGGTCTGACGCTCAGTGGAACGAAAACTCACGTTAAGGGATTTTGGTCATGAGATTATCAAAAAG

ColE1

7,400 7,420 7,440 7,460 7,480

GATCTTCACCTAGATCCTTTTGGATC

ColE1

7,500 7,510
